# Exercise and cancer-related fatigue in adults: a systematic review of previous systematic reviews with meta-analyses

**DOI:** 10.1186/s12885-017-3687-5

**Published:** 2017-10-23

**Authors:** George A. Kelley, Kristi S. Kelley

**Affiliations:** 1 Meta-Analytic Research Group, School of Public Health, Department of Biostatistics, Director, WVCTSI Clinical Research Design, Epidemiology, and Biostatistics (CRDEB) Program, PO Box 9190, Robert C. Byrd Health Sciences Center, Room 2350-A, Morgantown, West Virginia 26506-9190 USA; 2 Meta-Analytic Research Group, School of Public Health, Department of Biostatistics, PO Box 9190, Robert C. Byrd Health Sciences Center, Room 2350-B, Morgantown, West Virginia 26506-9190 USA

**Keywords:** Exercise, Cancer, Fatigue, Meta-analysis, Systematic review

## Abstract

**Background:**

Conduct a systematic review of previous systematic reviews with meta-analysis to determine the effects of exercise (aerobic, strength or both) on cancer-related-fatigue (CRF) in adults with any type of cancer.

**Methods:**

Systematic reviews with meta-analyses of previous randomized controlled trials published through July of 2016 were included by searching six electronic databases and cross-referencing. Dual-selection and data abstraction were conducted. Methodological quality was assessed using the Assessment of Multiple Systematic Reviews (AMSTAR) instrument. Standardized mean differences (SMD) that were pooled using random-effects models were included as the effect size. In addition, 95% prediction intervals (PI), number needed-to-treat (NNT) and percentile improvements were calculated.

**Results:**

Sixteen studies representing 2 to 48 SMD effect sizes per analysis (mean ± SD, 7 ± 8, median = 5) and 37 to 3254 participants (mean ± SD, 633 ± 690, median = 400) were included. Length of training lasted from 3 to 52 weeks (mean ± SD, 14.6 ± 3.1, median = 14), frequency from 1 to 10 times per week (mean ± SD, 3.4 ± 0.8, median = 3), and duration from 10 to 120 min per session (mean ± SD, 44.3 ± 5.5, median = 45). Adjusted AMSTAR scores ranged from 44.4% to 80.0% (mean ± SD, 68.8% ± 12.0%, median = 72.5%). Overall, mean SMD improvements in CRF ranged from −1.05 to −0.01, with 22 of 55 meta-analytic results (52.7%) statistically significant (non-overlapping 95% CI). When PI were calculated for results with non-overlapping 95% CI, only 3 of 25 (12%) yielded non-overlapping 95% PI favoring reductions in CRF. Number needed-to-treat and percentile improvements ranged from 3 to 16 and 4.4 to 26.4, respectively.

**Conclusions:**

A lack of certainty exists regarding the benefits of exercise on CRF in adults. However, exercise does not appear to increase CRF in adults.

**Trial registration:**

PROSPERO Registration # CRD42016045405.

**Electronic supplementary material:**

The online version of this article (10.1186/s12885-017-3687-5) contains supplementary material, which is available to authorized users.

## Background

Cancer is the second leading cause of death in the world, accounting for approximately 8.7 million deaths in 2015 [1]. In addition, the number of cases in 2015 was estimated at 17.5 million, an increase of 13% since 2005 [1]. Furthermore, cancer was estimated to have resulted in 208.3 million disability-adjusted-life-years in 2015 [1]. Not surprisingly, the economic costs of cancer are high. For example, in 2009, it was estimated that the 12.9 million new cases of cancer worldwide cost approximately $286 billion for that year only [2]. For the estimated 21.5 million new cases expected in 2030, costs are projected to increase to approximately $458 billion [3].

Recent advances in the treatment of cancer have resulted in increased survival rates. For example, in the United States, the number of cancer survivors increased from 7 million in 1992 to more than 14 million in 2014, and is expected to increase to approximately 19 million by 2024 [4]. Given the increasing number of cancer patients and survivors, there will be a congruent increase in the number of cancer patients and survivors who will have to deal with the side effects of cancer treatment(s). One of the most significant side-effects is cancer-related-fatigue (CRF) [5, 6], a condition that is highly prevalent both during and after treatment. [5] While varying depending on the type of cancer and treatment, up to 91% of patients have reported experiencing CRF during treatment [7, 8]. The prevalence of CRF also remains high after treatment. For example, 35% and 34% of breast cancer survivors have reported CRF one to five years and five to ten years post treatment, respectively [9]. The effects of CRF also have deleterious effects on patients’ and survivors’ physical, mental, and emotional well-being [5].

The National Comprehensive Cancer Network’s (NCCN) Clinical Practice Guidelines in Oncology recommend physical activity as a nonpharmacologic strategy for the management of CRF both during and after treatment [6]. This includes both aerobic (walking, swimming, etc.) and resistance training, i.e., weight training exercises [6]. However, while a large number of systematic reviews with meta-analyses on exercise and CRF have been conducted, the direction of results and especially the magnitude of effect have varied substantially [10–36]. This is problematic because healthcare practitioners and decision makers who at one time relied on systematic reviews to guide practice and decision-making are now overwhelmed with multiple systematic reviews on exercise and CRF [10–36]. A plausible and more recently accepted approach for addressing these multiple reviews is to conduct a systematic review of previous systematic reviews so that the findings of these reviews can be assessed and compared using strict methodology [37]. In addition to guiding practice and decision-making, systematic reviews of previous systematic reviews with meta-analysis are important for improving the quality and reporting of future reviews of this nature as well as determining whether another systematic review with meta-analysis is warranted on the topic of interest [38]. Furthermore, such reviews can help provide direction for researchers conducting their own original research. Thus, given (1) multiple systematic reviews with meta-analysis on exercise and CRF in cancer patients and survivors, including the conflicting findings of such [10–36], (2) the need to systematically review multiple systematic reviews for both applied and research reasons [37], and (3) to the best of the authors’ knowledge, the nonexistence of any previous systematic review of systematic reviews with meta-analysis of randomized controlled trials on exercise and CRF, the purpose of the current study was to conduct a systematic review of previous systematic reviews with meta-analyses on exercise and CRF in adults.

## Methods

Where appropriate, this study was conducted according to the Preferred Reporting Items for Systematic Review and Meta-Analysis (PRISMA) Statement [39]. The protocol is registered in PROSPERO (Registration # CRD42016045405).

### Study eligibility

Studies were eligible for inclusion if they met all of the following a priori criteria: (1) adults 18 years of age and older who were cancer patients or cancer survivors with any type of cancer, (2) exercise (aerobic, strength training or both) lasting at least 3 weeks in length, (3) any measure of CRF as the primary outcome, (4) change outcome difference results between exercise and control group (nonintervention, usual care, attention control, wait-list control) for CRF reported, (5) systematic review with meta-analysis of randomized controlled trials or data reported separately for randomized controlled trials in which at least two studies were pooled, (6) published and unpublished studies (dissertations, master’s theses, etc.) at any time point and in any language. Exercise, aerobic exercise and strength training exercise were defined according to the 2008 US Physical Activity Guidelines Advisory Committee Report [40]. A priori, studies were limited to interventions lasting at least 4 weeks because of an interest in examining the chronic versus acute effects of exercise on CRF. However, a *post-hoc* decision was made to include systematic reviews and meta-analyses that included studies of at least 3 weeks since the benefits of exercise on CRF have been realized with interventions of this length [41].

Meta-analyses that pooled results for aerobic and/or resistance training along with meditative movement therapies such as yoga, tai chi and qi gong were also eligible for inclusion. However, if separate results were reported for aerobic and/or resistance training only, only those findings were included. Studies that only included meditative movement therapies were excluded because of the meditative component of these interventions. Meta-analyses were limited to those that pooled randomized controlled trials because randomized controlled trials are the only way to control for unidentified confounders as well as the fact that nonrandomized controlled trials tend to overestimate the effects of therapy in healthcare interventions [42, 43].

Studies were excluded based on any one of the following: (1) inappropriate population (study not limited to adults 18 years of age and older who were cancer patients or cancer survivors, etc.), (2) inappropriate intervention (nutrition intervention, exercise less than 3 weeks in length, etc.), (3) inappropriate comparison (change outcome difference between intervention and control group not calculated, exercise compared to nutrition, etc.), (4) inappropriate outcome (CRF not assessed as a primary outcome), (5) inappropriate study design (studies pooled in meta-analysis not limited to randomized controlled trials, etc.).

### Data sources

Studies were located by searching the following six electronic databases from their inception up to July, 2016: (1) PubMed, (2) Sport Discus, (3) Web of Science, (4) Scopus, (5) Cochrane Database of Systematic Reviews, and (6) ProQuest Dissertations and Theses. In addition, cross-referencing from retrieved meta-analyses were also searched for potentially eligible meta-analyses. While the exact search strategy varied slightly according to the requirements of each database, the search strategy was similar to that used for PubMed:

“(exercise OR physical fitness) AND (systematic review OR meta-analy*) AND (fatigue) AND cancer”.

All searches were conducted by the first author and initially stored in Reference Manager, version 12.0.3 [44]. However, since Reference Manager was no longer supported after December 31, 2016, all references were imported into EndNote X8 [45]. A copy of all database searches can be found in Additional file 1.

### Study selection

After electronic and manual removal of duplicates by the first author, all remaining studies were selected independently by both authors. They then met and reviewed their selections for agreement. Any disagreements were resolved by consensus. The overall precision of the searches was calculated by dividing the number of studies included by the total number of studies screened after removing duplicates [46]. The number needed to read (NNR) was then calculated as the inverse of the precision [46].

### Data abstraction

Prior to data abstraction, a codebook that could hold up to 278 items per study was developed, pilot-tested, and revised by both authors in Microsoft Excel 2013 [47]. The major categories of items coded included (1) study characteristics (author, year, journal, country study conducted, etc.), (2) participant characteristics (age, height, body weight, type of cancer, etc.), (3) intervention characteristics (length, frequency, intensity, duration, mode, compliance, etc.) and (4) outcome characteristics (sample size, number of effect sizes for CRF, effect size statistics for CRF, type of CRF assessed, etc.). All studies were coded by both authors, independent of each other. They then met and reviewed every item for agreement. Any disagreements were resolved by consensus. Cohen’s kappa statistic (*κ*) was used to measure inter-rater agreement prior to correcting discrepant items [48].

### Evaluation of systematic reviews included

Each included systematic review with meta-analysis was evaluated using the Assessment of Multiple Systematic Reviews (AMSTAR) Instrument, an 11-item instrument designed to assess the quality of systematic reviews and previously shown to be both valid and reliable [49]. Responses are coded as either “yes”, “no”, “can’t answer” or “not applicable”. “Can’t answer” is chosen when an item is applicable but not described by the authors. “Not applicable” is selected when an item is not applicable, for example if a systematic review was conducted but no meta-analysis was possible. For consistency when summing responses, the question “Was the status of publication (i.e. grey literature) used as an inclusion criterion?” was modified to “Was the status of publication (i.e. grey literature) used as an inclusion criterion avoided?” Assessments were conducted by both authors, independent of each other. They then met and reviewed every item for agreement. Any disagreements were resolved by consensus.

To evaluate the potential impact of each included study, the total frequency that each included systematic review with meta-analysis was cited as well as the mean number of citations each year was calculated. This was estimated using version 5.24 of Publish or Perish (Google Scholar Citation mechanism) [50]. In addition, the journal impact factor for the year that each study was published was also abstracted using Journal Citation Reports®.

### Data synthesis

Results for CRF from each original meta-analysis were coded with a concentration on random-effects models given that between-study heterogeneity is incorporated into the model [51, 52]. For those studies that reported results using a fixed-effect model, results were recalculated using the random-effects model of Dersimonian and Laird [53]. For each meta-analysis that included at least two effect sizes, the standardized mean difference (SMD), 95% confidence intervals (CI), z value, alpha value for z, Q statistic for heterogeneity [54], *I* ^*2*^ statistic for inconsistency and tau-squared (*τ* ^2^) were extracted or calculated if sufficient data were available [55]. If results were presented in graphical format and numerical data were not available, they were estimated using WebPlotDigitizer (version 3.8) [56]. A two-tailed alpha value ≤0.05 for z and non-overlapping 95% CI were considered to represent statistically significant SMD changes in CRF. For the Q statistic, an alpha value ≤0.10 was considered statistically significant. I-squared values of 0% to <25%, 25% to <50%, 50% to <75% and ≥75% were considered to represent low, moderate, large, or very large amounts of inconsistency [55]. Data for small-study effect results (publication bias, etc.) [57], were also extracted or calculated if adequate data were available. If possible, small-study effects was analyzed using the regression-intercept approach of Egger et al. [57, 58], assuming there were at least 10 effect sizes [57]. One-tailed 95% CIs that did not include zero (0) were reflective of statistically significant small-study effects. To avoid violating the assumption of independence, a decision was made a priori to not pool results from the different meta-analyses into one overall result based on the expectation that one or more of the same randomized controlled trials would be included in the different meta-analyses. Since it was also assumed, a priori, that none of the included meta-analyses would report 95% prediction intervals (PIs) [59–61], these were calculated if the findings of the original meta-analyses were statistically significant and the data from each included study from each meta-analysis were available [59–61]. Prediction intervals are calculated for the purpose of estimating the treatment effect in a new study [59–61], and have been suggested to be preferable to 95% CI for decision analysis [62].

To reinforce practical application and under the a priori assumption that none of the studies would report such data, the number-needed-to treat (NNT) [63] and Cohen’s U_3_ index for percentile improvement [64] were also calculated for those findings reported as statistically significant. For NNT, the method of Kraemer and Kupfer [63] was used versus a method based on control group risk given the lack of consensus regarding an appropriate control group risk for CRF. For Cohen’s U_3_ index [64], a SMD of 0.30, for example, suggests that exercise group participants would be at approximately the 62nd percentile with respect to reducing their fatigue [65]. This equates to exercise group participants being approximately 12 percentiles higher than control group participants [65].

The percentage of yes responses for AMSTAR results were calculated for each study and included both unadjusted scores as well as scores adjusted for “not applicable” and “cannot answer” responses. A Pearson correlation coefficient was used to examine the association between adjusted and unadjusted AMSTAR scores with the impact factor of the journal from which the study was published. A two-tailed probability value ≤0.05 was considered statistically significant. All analyses for the current study were conducted using Microsoft Excel 2013 [47], and MetaXL (version 5.3) [66].

## Results

### Characteristics of included meta-analyses

**Fig. 1:**
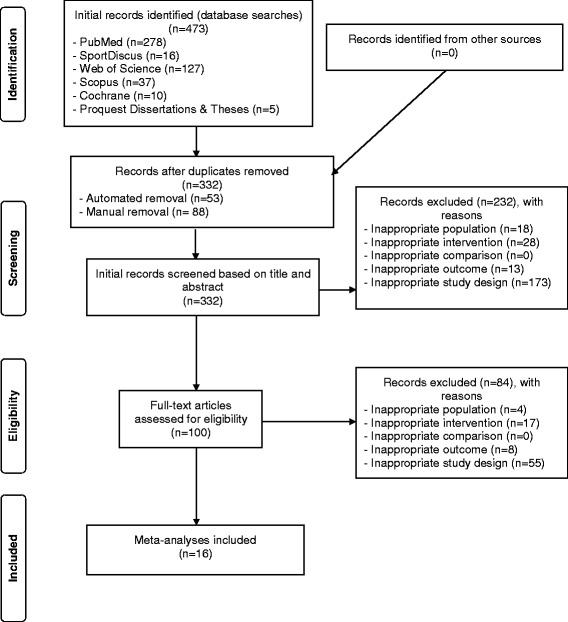
Flow diagram for the selection of studies

Of the 332 non-duplicate records reviewed, 16 aggregate data meta-analyses met the criteria for inclusion [10–13, 16–19, 24–26, 31, 33–36]. The precision of the search was 0.05 while the NNR was 21. A flow diagram that depicts the search and selection process is shown in Fig. 1 while the general characteristics of each included meta-analysis is shown in Table 1. The included studies were published between 2007 and 2016 (mean ± SD, 2013 ± 2.8, median = 2014). For those studies that were excluded, the primary reasons for omission were inappropriate study design (72.2%), intervention (14.2%), population (7.0%), and outcomes (6.6%). A list of excluded studies, including the reasons for exclusion, can be found in Additional file 2. Journal impact factors for included studies ranged from 1.6 to 17.2 (mean ± SD, 4.8 ± 4.2, median = 3.3). Fourteen of the 16 meta-analysis (87.5%) reported receiving funding for their work [10–13, 16–19, 24, 25, 31, 33, 34, 36]; 4 from either university [10, 24, 25, 31] or private [13, 16, 17, 34] sources, 3 from both government and private sources [11, 12, 19] and 2 from government sources only [18, 36]. All 10 meta-analyses (62.5%) in which data were available reported no competing interests [10, 11, 16, 17, 24–26, 31, 34, 36]. Only 2 (12.5%) reported registering the protocol for their systematic review with meta-analysis [25, 26], both in PROSPERO, an international prospective register of systematic reviews with or without meta-analysis.

**Table 1:** General characteristics of included meta-analyses^a^

Reference	Year	Country	Studies	Participants	Sex (F/M)	Cancer Types	Interventions	CRF Assessment
Brown et al. [10]	2011	United States	44	3254	F/M	breast, colorectal, leukemia, lymphoma, prostate, mixed	AE and/or ST	BFI, EORTC-QLQ C30, FACT, FACT-An, FACT-B, FACT-F, FACT-G, LAS, PFS, POMS, SAS, SCFS, SF-36, 0–9 scale
Carayol et al. [11]	2013	France	11	–	F	breast	AE and/or ST	BFI, FACT-F, MFI, PFS, SF-36(Vitality)
Carayol et al. [12]	2015	France	21	2181	F	breast	AE and/or ST	SF-36(Vitality)
Cramer et al. [13]	2014	Germany	3	157	F/M	colorectal	AE and/or ST	FACT-F
Duijts et al. [16]	2011	Netherlands	11	1244	F	breast	AE and/or ST	FACT-F, FS, LAS-F, POMS, PFS, SCFS
Fong et al. [17]	2012	China	6	758	F/M	breast, colon, colorectal, endometrial, gastric, gynecological, lung, lymphoma, testicular	AE and/or ST	EORTC-F, FACT-F, PFS
Jacobsen et al. [18]	2007	United States	12	833	F/M	breast, colon, colorectal, multiple myeloma, prostate, mixed	AE and/or ST	–
Kangas et al. [19]	2008	United States	16	1001	F/M	breast, colorectal, gastrointestinal, lung, myeloma, prostate, mixed	AE and/or ST	BFI, EORTC QLQ C30, FACIT-F, FACT-B, FACT-C, FACT-P/F, FACT-B/G, LAS-F, LASA fatigue/energy/anxiety/depression, PFS, PFS-R, POMS, POMS-F, POMS-SV, SAS-F, SF-36
Meneses-Echavez et al. [24]	2015	Colombia	9	–	F/M	breast, prostate, mixed	AE and/or ST	EORTC QLQ C30, FACT-B, FACT-F, FACT-G, FACT-P, PFS, SCFS
Meneses-Echavez et al. [25]	2015	Colombia	9	1016	F	breast	AE and/or ST	EORTC QLQ C30, FACT-B, FACT-F, FACT-G, FACT-P, PFS, SCFS
Meneses-Echavez et al. [26]	2015	Colombia	11	1427	F/M	breast, lymphoma, prostate	AE and/or ST	EORTC QLQ C30, FACT-B, FACT-F, FACT-G, FACT-P, PFS, SCFS
Tian et al. [31]	2016	China	26	2830	F/M	breast, colorectal, gynecologic, hematological, nasopharyngeal prostate, other	AE	BFI, FACT-F, LASA, PFS, PFS-R, POMS
Van Haren et al. [33]	2013	Netherlands	2	115	–	cancer patients undergoing hematopoietic stem cell transplantation	AE and/or ST	FACT-An, MFI, POMS-F
Van Vulpen et al. [34]	2016	Netherlands	3	–	F/M	breast	AE and/or ST	FAQ, MFI-20
Velthuis et al. [35]	2010	Netherlands	7	674	F/M	acute myelogenous leukemia, breast, multiple myeloma, prostate	AE and/or ST	BFI, FACIT-F, FACT-An, FACT-F, PFS, PFS-R, POMS, SAS
Zou et al. [36]	2014	China	6	371	–	breast	AE	FACIT-F, PFS-R

Notes: ^a^Data representative of studies in which CRF was assessed; −-, data not available; *CRF* Cancer-Related Fatigue, *F* is Female and *M* is Male, *AE* aerobic exercise, *ST* Strength training, *BFI* Brief Fatigue Index, *EORTC-F* European Organization for Research and Treatment of Cancer – Fatigue, *EORTC* QLQ-C30, European Organization for Research and Treatment of Cancer Quality of Life Questionnaire; *FACIT-F* Functional Assessment of Chronic Illness Therapy – Fatigue scale, *FACT* Functional Assessment of Cancer Therapy, *FACT-An* Functional Assessment of Cancer Therapy – Anemia, *FACT-B* Functional Assessment of Cancer Therapy – Breast Cancer, *FACT-B/G* Functional Assessment of Cancer Therapy – Breast Cancer/General, *FACT-C* Functional Assessment of Cancer Therapy – Colon Cancer, *FACT-F* Functional Assessment of Cancer Therapy – Fatigue, *FACT-G* Functional Assessment of Cancer Therapy – General, *FACT-P/F* Functional Assessment of Cancer Therapy – Prostate Cancer and Fatigue, *FAS* Fatigue Assessment Questionnaire, *LAS* Linear Analog Scale, *LASA* Linear Analog Self-Assessment, *LASA-F* Linear Analog Self-Assessment-Fatigue, *MFI* Multidimensional Fatigue Inventory, *PFS* Piper Fatigue Scale, *PFS-R* Piper Fatigue Scale-Revised, *POMS* Profile of Moods States, *POMS-SV* Profile of Mood States, Short-Version, *SAS* Symptom Assessment Scale, *SAS-F* Symptom Assessment Scale-Fatigue, *SCFS* Schwartz Cancer Fatigue Scale, *SF-36* Medical Outcomes Study, Short-Form 36

With respect to country, 4 were conducted in the Netherlands [16, 33–35], 3 in either China [17, 31, 36], Colombia (by the same research group) [24–26], or the United States [10, 18, 19], 2 in France [11, 12] and 1 in Germany [13]. The number of studies nested in each meta-analytic study that assessed CRF ranged from 2 to 44 (mean ± SD, 12 ± 11, median = 10) while the total number of participants ranged from 115 to 3254 (mean ± SD, 1220 ± 980, median = 1001) for the 13 studies in which data were provided or could be calculated [10, 12, 13, 16–19, 25, 26, 31, 33, 35, 36]. Dropout data were reported as an average of 19.1% in one meta-analysis [35], less than 15% for more than 50% of included studies in two meta-analyses by the same research group [11, 12], and less than 15% for 88.9% of studies included in another meta-analysis [24].

Risk of bias/study quality for the studies included in each meta-analytic study was assessed using the Physiotherapy Evidence Database (PEDro) scale in 7 studies (43.4%) [10–12, 24–26, 35], the Cochrane Risk of Bias Assessment Instruments in 6 studies (37.5%) [12, 13, 18, 31, 33, 34], the Newcastle Ottawa Scale [36], Consort [19] and Delphi [19] checklists, Quality Assessment Checklist developed by the Scottish International Guidelines Network [17], and the Newall instrument [18]. Mean scores from the most commonly used instrument (PEDro) ranged from 58.0% to 70.0%.

For the 14 (87.5%) meta-analyses in which data were available [10–13, 16–19, 24–26, 31, 34, 35], 10 (62.5%) included studies that consisted of males and/or females [10, 13, 17–19, 24, 26, 31, 34, 35] while 4 (25%) were limited to females [11, 12, 16, 25]. Three meta-analyses (18.8%) in which sufficient data were available reported the inclusion of studies representing multiple races and ethnicities [25, 31, 36]. Eight of the 16 meta-analyses (50.0%) included participants with multiple types of cancer [10, 17–19, 24, 26, 31, 35] while 6 (37.5%) were limited to breast cancer [11, 12, 16, 25, 34, 36], 1 to colorectal cancer [13], and 1 to cancer patients undergoing hematopoietic stem cell transplantation [33]. Fourteen of the 16 meta-analyses (87.5%) included studies in women with breast cancer [10–12, 16–19, 24–26, 31, 34–36], followed by prostate (43.8%) [10, 18, 19, 24, 26, 31, 35] and colorectal (37.5%) [10, 13, 17–19, 31] cancer. Other types of cancer included multiple myeloma [18, 19, 35], lymphoma [10, 17, 26], lung [17, 19], colon [17, 18], leukemia [10, 35], gynecologic [17, 31], gastrointestinal [17, 19], endometrial [17], testicular [17], nasopharyngeal [31], and hematological [31]. For the 10 meta-analyses (62.5%) that provided information [11, 13, 16, 18, 19, 24–26, 31, 35] cancer stages of participants in the included studies ranged from what was defined as early to stage IV as well as Duke’s Stage A through C, any, and 1–3 for colorectal cancer [13]. Eight meta-analyses (50.0%) included studies in which participants were either currently or previously receiving cancer treatment [10, 13, 16, 18, 19, 24, 25, 31] while 7 (43.8%) were limited to those currently receiving treatment [11, 12, 26, 33–36]. One other meta-analysis was limited to studies in which participants were previously treated [17].

Fourteen (87.5%) of the meta-analyses included studies in which aerobic and resistance training, either alone or in combination, were performed [10–13, 16–19, 24–26, 33–35]. Two other meta-analyses (12.5%) focused on studies in which aerobic exercise was performed [31, 36]. Length of training for the included studies in each meta-analysis ranged from 3 to 52 weeks (mean ± SD, 14.6 ± 3.1, median = 14), frequency from 1 to 10 times per week (mean ± SD, 3.4 ± 0.8, median = 3), and duration from 10 to 120 min per session (mean ± SD, 44.3 ± 5.5, median = 45). Intensity of training for aerobic exercise was reported using a variety of methods. These included metabolic equivalents (METS) [10–12], the Borg scale [34], percentage of maximum heart rate [24–26, 31, 35], maximum heart rate reserve [31, 35], and maximum oxygen consumption (VO_2max_) [13, 31, 35]. For strength training, intensity was reported as one-repetition maximum (1 RM) [34, 35] or as METS [10]. Categorically, aerobic and strength training intensities for studies included in the meta-analyses represented light, moderate and vigorous exercise [67]. Compliance for the studies included in each meta-analysis and defined as the percentage of exercise sessions attended ranged from 16% to 100% (mean ± SD, 68.7 ± 18.5) [12] and 71% to 83% (mean ± SD, 76.0 ± 6.0) [34] for the 2 studies reporting this type of information. Another 2 meta-analyses reported compliance as greater than 60% for more than 50% of the included studies [11] and greater than 80% for 9 studies and less than 80% for 11 studies they included [31].

Assessment of CRF from the studies included in each meta-analysis was accomplished using a variety of instruments. The two most commonly reported instruments were the Functional Assessment of Cancer Therapy scales (75.0% of meta-analyses) [10, 11, 13, 16, 17, 19, 24–26, 31, 33, 36], and the Piper Fatigue scales (68.8% of meta-analyses) [10, 11, 16, 17, 19, 24–26, 31, 35, 36]. For adverse events, five (31.3%) of the included meta-analyses provided information about adverse events from the studies they included [24–26, 31, 35]. Three meta-analyses by the same research group reported that 2 of 9 studies (22.2%) in each of two meta-analyses reported information on adverse events [24, 25], while a third reported that 3 studies (27.0%) reported data on adverse events [26]. A fourth meta-analysis reported that 12 of 26 included studies (46.2%) reported adverse events but none were directly related to the study [31] while a fifth reported that 12 of 18 included studies (67.0%) reported information on adverse events [35]. Finally, none of the included meta-analyses reported any information about the costs of the interventions from the studies they included [10–13, 16–19, 24–26, 31, 33–36].

### Methodological quality and impact

Itemized results for each study based on the AMSTAR instrument can be found in Additional file 3 **.** Unadjusted scores ranged from 36.4% to 72.7% (mean ± SD, 59.1% ± 11.5%, median = 63.6%) while adjusted scores ranged from 44.4% to 80.0% (mean ± SD, 68.8% ± 12.0%, median = 72.5%). All studies included an a priori design and study characteristics Table [10–13, 16–19, 24–26, 31, 33–36], while all but one (93.8%) reported adequate information regarding the assessment of study quality [10–13, 17–19, 24–26, 31, 33–36], using study quality findings in formulating conclusions [10–13, 17–19, 24–26, 31, 33–36], and using appropriate methods to combine the results of studies [10–13, 16, 17, 19, 24–26, 31, 33–36]**.** Seven of the studies clearly reported dual study selection and data extraction procedures [13, 17, 19, 24, 25, 34, 35]. None of the meta-analyses provided a reference list of excluded studies and the reason(s) for exclusion nor did they include information regarding conflict of interest from the studies they included [10–13, 16–19, 24–26, 31, 33–36]. Eleven studies (68.8%) assessed small-study effects (publication bias, etc.) [10–12, 16, 17, 19, 24, 25, 33, 34, 36], six clearly performed a comprehensive literature search [10, 12, 13, 17, 26], while three avoided the status of publication as an inclusion criterion [24–26]. There was no statistically significant association between the overall AMSTAR score and journal impact factor for either unadjusted (*r* = 0.298, *p* = 0.26) or adjusted (*r* = 0.163, *p* = 0.55) values.

With respect to impact, the total number of times that each meta-analysis was cited across all years ranged from 6 to 296 (mean ± SD, 97 ± 107, median = 30). When adjusted for the number of years that each meta-analysis was available, the number of times that each meta-analysis was cited per year ranged from 6 to 74 (mean ± SD, 22 ± 18, median = 17). Across all years and meta-analyses, the total number of citations was 1554 while the citation rate per year was 357.

### Data synthesis

#### Overall findings

**Fig. 2:**
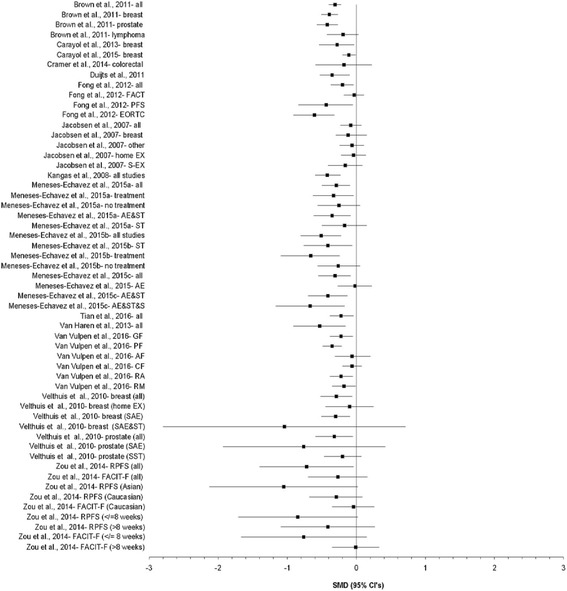
Forest plot for standardized mean difference effect size changes in CRF based on confidence intervals. The black squares represent the pooled standardized mean difference effect size for each analysis while the left and right extremes of the squares represent the corresponding 95% confidence intervals for the pooled standardized mean difference (SMD) effect size for each analysis. All analyses are based on a random-effects model and not pooled across all analyses because some of the results included the same studies. AE&ST&S, Aerobic exercise, strength training and stretching; AE&ST, Aerobic exercise and strength training; AF, Affective fatigue; CF, Cognitive fatigue; EORTC, European Organization for Research and Treatment of Cancer; EX, Exercise; FACT, Functional Assessment of Cancer Therapy; GF, General fatigue; PF, Physical fatigue; PFS, Piper Fatigue Scale; RA, Reduced activity; RM, reduced motivation; SAE&ST, Supervised aerobic exercise and strength training; SAE, Supervised aerobic exercise; SST, Supervised strength training; ST, Strength training

**Fig. 3:**
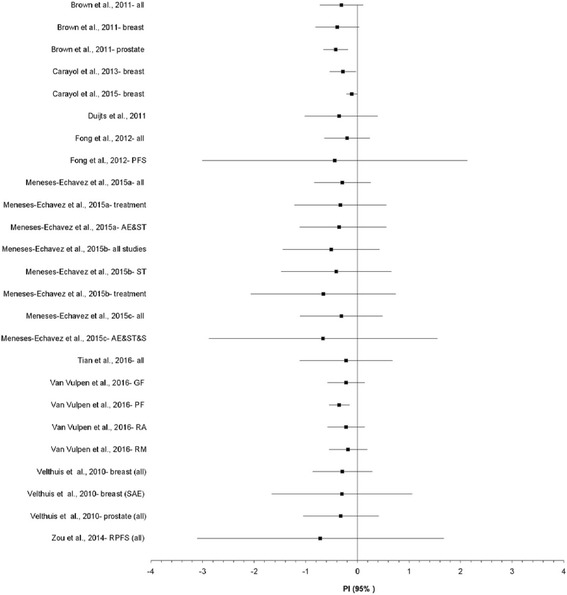
Forest plot for standardized mean difference effect size changes in CRF based on prediction intervals. The black squares represent the pooled standardized mean difference (SMD) effect size for each analysis while the left and right extremes of the squares represent the corresponding 95% prediction intervals, derived from the SMD and 95% confidence intervals for each analysis. All analyses are based on a random-effects model and limited to those that were statistically significant (*p* ≤ 0.05) with non-overlapping 95% confidence intervals. Results were not pooled across all analyses because some of the results included the same studies. AE&ST&S, Aerobic exercise, strength training and stretching; AE&ST, Aerobic exercise and strength training; GF, General fatigue; PF, Physical fatigue; PFS, Piper Fatigue Scale; RA, Reduced activity; RM, reduced motivation; SAE, Supervised aerobic exercise; ST, Strength training

Results for changes in CRF based on confidence intervals and prediction intervals are shown in Figs. 2 and 3 respectively, while detailed results for both are shown in Additional file 4. A total of 55 analyses from the 16 studies were included [10–13, 16–19, 24–26, 31, 33–36]. The number of SMD effect sizes in each analysis ranged from 2 to 48 per analysis (mean ± SD, 7 ± 8, median = 5) while the number of participants nested within each of the 41 analyses in which data were available ranged from 37 to 3254 (mean ± SD, 633 ± 690, median = 400). In addition to overall results, the authors of these previous meta-analyses reported subgroup analyses that included, but were not limited to, type of cancer [10, 18, 35], instrument used to assess CRF [17, 36], component of CRF [34], whether participants were currently receiving treatment for cancer [24, 25], race/ethnicity [36], as well as various characteristics of the exercise interventions (type of exercise, home versus supervised, length) [18, 24–26, 35, 36].

**Fig. 4:**
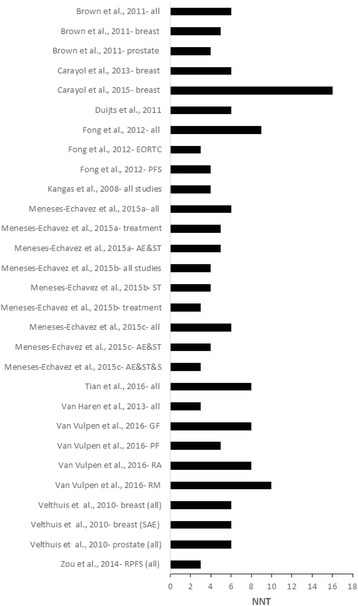
Horizontal bar graph for NNT. Numbers were derived from the pooled standardized mean difference (SMD) effect size for each meta-analysis and based on a random-effects model in which results were statistically significant (p ≤ 0.05) with non-overlapping 95% confidence intervals. Results were not pooled across all analyses because some of the results included the same studies. AE&ST&S, Aerobic exercise, strength training and stretching; AE&ST, Aerobic exercise and strength training; EORTC, European Organization for Research and Treatment of Cancer; GF, General fatigue; PF, Physical fatigue; PFS, Piper Fatigue Scale; RA, Reduced activity; RM, reduced motivation; SAE, Supervised aerobic exercise; ST, Strength training

**Fig. 5:**
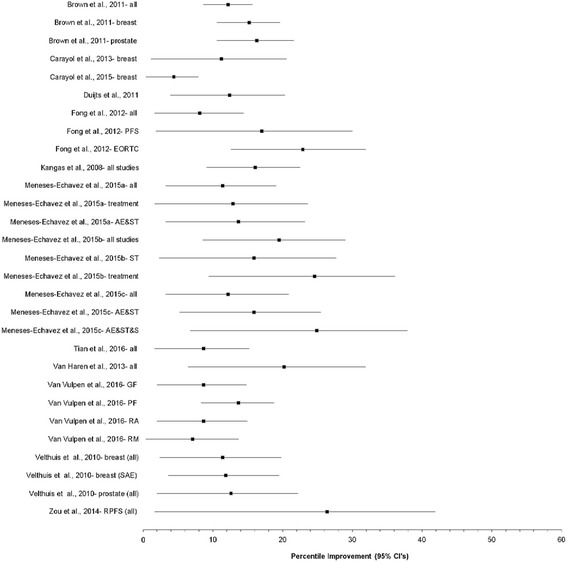
Forest plot for percentile improvements in fatigue. The black squares represent the pooled percentile improvement for each analysis while the left and right extremes of the squares represent the corresponding 95% confidence intervals for percentile improvement for each analysis. Values were derived from the pooled standardized mean difference (SMD) effect size for each meta-analysis and based on a random-effects model in which results were statistically significant (*p* ≤ 0.05) with non-overlapping 95% confidence intervals. Results were not pooled across all analyses because some of the results included the same studies. AE&ST&S, Aerobic exercise, strength training and stretching; AE&ST, Aerobic exercise and strength training; EORTC, European Organization for Research and Treatment of Cancer; GF, General fatigue; PF, Physical fatigue; PFS, Piper Fatigue Scale; RA, Reduced activity; RM, reduced motivation; SAE, Supervised aerobic exercise; ST, Strength training

Overall, mean SMD improvements in CRF ranged from −1.05 to −0.01. Twenty-nine of 55 meta-analytic results (52.7%) were statistically significant with non-overlapping 95% CI. More than half of the statistically significant findings (57.1%) yielded statistically significant heterogeneity based on the Q statistic while inconsistency, i.e., *I* ^*2*^ *,* ranged from 0% to 89% for those results that were statistically significant. When PI were calculated for those analyses in which data were available and were statistically significant, only 3 of 25 (12%) yielded non-overlapping 95% PI favoring reductions in CRF. Tau-squared values for PI ranged from 0 to 0.61. The NNT based on the mean SMD for each statistically significant meta-analysis ranged from 3 to 16 (Fig. 4 and Additional file 5) while percentile improvements ranged from 4.4 to 26.4 (Fig. 5 and Additional file 5).

For breast cancer, the most common type of cancer investigated (50.9% of all analyses), overall mean SMD improvements in CRF ranged from −1.05 to −0.01. Fourteen of 28 meta-analytic (50.0%) results were statistically significant with non-overlapping 95% CI. More than half of the statistically significant findings (57.1%) yielded statistically significant heterogeneity while inconsistency, i.e., *I* ^*2*^ *,* ranged from 0% to 89% for those results that were statistically significant. When PI were calculated for those analyses in which data were available and statistically significant, only 2 of 14 (14.3%) yielded non-overlapping 95% PI favoring reductions in CRF. Tau-squared values for PI ranged from 0 to 0.61. The NNT for CRF based on the mean SMD for each statistically significant breast cancer meta-analysis ranged from 3 to 16 while percentile improvements ranged from 4.4 to 26.4.

#### Small study effects, influence analysis and cumulative meta-analysis

Four studies reported potential *small-study effects* [16, 24, 25, 31] while another 7 reported no such effects [10–12, 17, 19, 34, 36]. *Influence analysis* for overall results within each meta-analysis and in which data could be calculated ranged from −0.64 to −0.05 while c*umulative meta-analysis,* ranked by year, yielded findings that stabilized and remained statistically significant between 2001 and 2013.

#### Other results reported by investigators of original meta-analyses

Brown et al. [10], reported results that were limited to one SMD in which no statistically significant changes in CRF were observed for either colorectal cancer or leukemia. In addition, across all types of cancers, neither session length in minutes, number of exercise sessions, nor treatment with radiation therapy were shown to be statistically significant moderators of changes in CRF [10]. When results for resistance training were partitioned according to light and moderate intensity exercise and further partitioned according to whether theory was used in guiding the interventions, statistically significant improvements in CRF were found for all categories except light intensity activity in which no theory was used in planning the intervention [10]. Statistically significant improvements in CRF were also found for both light and moderate intensity resistance training across all three age categories (39, 65 and 70 years of age) as well as across all three categories of study quality except light intensity training at the highest level of study quality [10].

Carayol et al., reported statistically significant improvements in CRF across all studies as well as when outliers were deleted [12]. Greater reductions in CRF were associated with less than 75% of the study sample currently receiving chemotherapy as well as less than 140 metabolic equivalent (METS) hours of prescribed exercise [12]. In addition, greater reductions in CRF were associated with meditative movement therapies (yoga, tai chi and qi gong) versus non-meditative movement therapies (aerobic and/or resistance exercise) [12].

A meta-analysis by Kangas et al., that was not limited to exercise interventions but reported data for such performed an extraordinarily large number of subgroup analyses that were limited to a small number of studies and effect sizes for each analysis [19].

Three very similar meta-analyses by Meneses-Echavez et al. were published within a two-year period [24–26]. One reported that length, frequency and duration were associated with improvements in CRF but not year of publication or training intensity [24]. Another similar meta-analysis reported statistically significant associations with length, frequency, duration and year of publication but not training intensity. [25] A third meta-analysis by Meneses-Echavez et al. [26], reported that one study limited to strength training showed a statistically significant benefit on CRF. Because of an apparent data entry error for at least one of the studies in their original meta-analysis, the large overall pooled results reported (SMD, −1.69, 95% CI, −2.99 to −0.39) as well as results for the aerobic exercise subgroup (SMD, −2.99, 95% CI, −6.49 to 0.51) were recalculated by the current investigative team by retrieving the original study [68] and then rerunning both analyses (see Fig. 2 and Additional file 4). The recalculated SMD and 95% CI for that study matched the results reported in the meta-analysis by Tian et al. [31] (SMD, 0.00, 95% CI, −0.18 to 0.18), a value much smaller than that reported by the original investigators (SMD, −15.14, 95% CI, −16.10, −14.19).

In a meta-analysis by Tian et al. [31], *s*tatistically significant reductions in CRF were found for off-treatment patients, those with nasopharyngeal carcinoma, breast cancer, professionally led aerobic exercise, walking, and home-based exercise.

Van Vulpen et al. [34], reported statistically significant reductions in CRF when limited to supervised exercise interventions as well as general fatigue and physical fatigue, but not cognitive fatigue or reduced activity and motivation.

Velthuis et al. [35], reported that supervised resistance training that was limited to one study resulted in a non-significant decrease in CRF in breast cancer patients while another study reported a nonsignificant decrease in CRF as a result of home-based exercise.

In the meta-analysis by Zou et al. [36], the authors reported that they analyzed all data using a random-effects model. However, when the current investigative team recalculated and pooled their findings using both a random-effects and fixed-effect model, it was apparent that the authors actually reported their findings using a fixed-effect model for at least five of the analyses. Recalculation of all analyses using the random-effects model of Dersimonian and Laird [53] reduced the magnitude of effect for all five analyses and reversed originally reported statistically findings to one of non-significance for the Revised Piper Fatigue Scale (RPFS) in Asians analysis. One study in the meta-analysis by Zou et al. [36], and limited to Asians resulted in statistically significant improvements in CRF when assessed using the Functional Assessment of Chronic Illness Treatment-Fatigue (FACIT-F) scale [36]. Univariate and multivariate meta-regression analyses, stratified by assessment type (RPFS and FACIT-F) and which included publication year, country, ethnicity and exercise time, resulted in no statistically significant associations [36]. Influence analysis with each study deleted from the model once resulted in no one study having a statistically significant effect on the overall findings [36].

## Discussion

### Findings

The purpose of the current study was to conduct a systematic review of previous systematic reviews with meta-analysis of randomized controlled trials regarding the effects of exercise (aerobic, strength training or both) on CRF in adults [10–13, 16–19, 24–26, 31, 33–36]. To the best of the authors’ knowledge, this is the first-ever comprehensive review on exercise and CRF. A large number of previous meta-analyses met the eligibility criteria (*N* = 16) and included a wide variety of (1) participant characteristics (age, gender, type of cancer, stage of cancer, treatment status, co-morbidities, etc.), (2) fatigue assessment instruments, and (3) intervention characteristics (type, length, frequency, intensity, duration, supervision, etc.). While the overall results of exercise on CRF varied substantially, all mean SMD effect sizes were in the direction of benefit. However, approximately 43% resulted in overlapping 95% confidence intervals, suggesting caution in any definitive, all-encompassing conclusions regarding the benefits of exercise on CRF. Prediction interval results warrant even greater caution when considering exercise in the treatment of CRF for at least two reasons. First, 88% of the PI overlapped. Second, from a practical perspective, PI may be more relevant than confidence intervals because they represent true effects while *I* ^*2*^ represents observed effects [69]. Results for meta-analyses focused on breast cancer, the most common cancer outcome studied, yielded similar results. Thus, improvements in CRF as a result of exercise are not only unimpressive, but also need to be taken into consideration when interpreting NNT and percentile improvement findings. Despite these inconclusive findings, it is noteworthy that none of the meta-analyses resulted in statistically significant increases in CRF. From the authors’ perspective, this is important given the possible perception in cancer patients that exercise may increase fatigue.

While a wide variety of subgroup and meta-regression analyses were performed above and beyond the overall pooled analyses, with some statistically significant and others inconclusive, it is important to realize that since studies are not randomly assigned to covariates in meta-analysis, they do not support causal inferences. Rather, they produce hypotheses about possible sources of heterogeneity and disparate effects that can be tested in future original studies [70]. Thus, the wide variety of subgroup and meta-regression analyses from these previous meta-analyses should be tested in a sufficiently powered randomized controlled trial. Finally, while it is generally considered that higher quality studies are published in journals with better, i.e., higher, journal impact factors, we found no association between such based on the AMSTAR instrument. One possible explanation for this is that factors other than study quality (for example, number of times an article is cited) are included when calculating journal impact factors [71–75].

The mechanisms by which exercise reduces cancer-related fatigue are not well established. Broadly, exercise may protect from treatment-related increases in CRF as opposed to reducing fatigue in patients upon treatment completion [29]. More specifically, exercise may reduce CRF by increasing cardiorespiratory fitness and muscle function [76]. LaVoy et al., suggested that the potential mechanisms by which exercise might reduce CRF include (1) improving psychological well-being and physical fitness, (2) decreasing inflammation, for example, increasing the level of anti-inflammatory cytokines, (3) improving autonomic nervous system function, for example increasing heart rate variability and thereby helping to restore balance between sympathetic and parasympathetic activity, and (4) neurotrophic factors, i.e., improved brain function [77].

### Implications for research

#### Implications for meta-analytic research

The results of the current investigation have at least five implications with respect to the reporting and conduct of future meta-analytic research. First, there is a need for clear and transparent reporting of future systematic reviews with meta-analysis so that such work may be replicated. For example, while recommended by the PRISMA guidelines [39], none of the meta-analyses included a complete reference list of excluded studies along with the reasons for exclusion.

Second, less than 13% of the included studies reported registering their systematic review with meta-analysis protocol in a data repository such as PROSPERO [25, 26]. The reasons for registering systematic reviews with or without meta-analysis are important and similar to those for registering randomized controlled trials. These include, but are not necessarily limited to: (1) avoidance of duplication, (2) the selective reporting of outcomes based on the direction of findings, and (3) greater transparency in what the original plan was for conducting the study. In addition to registering a systematic review, and similar to protocols for randomized controlled trials, some journals such as *Systematic Reviews* and *BMJ Open* now publish the protocols for systematic reviews, with or without meta-analysis, after undergoing peer review and being deemed acceptable.

Third, there is a need for accurate reporting of findings. For example, we found apparent errors in the calculation of effect sizes for one study [68] as well as errors in the reporting of the model used to pool results for another [36]. As was demonstrated, such errors can have a significant effect on the magnitude and direction of findings. Greater attention to detail on the part of investigators, reviewers and editors can help circumvent this issue.

Fourth, since some of the included meta-analyses may have been underpowered to find a true and meaningful effect, the inclusion of a trial sequential analysis (TSA) approach may be appropriate in future meta-analytic work so as to reduce Type I and Type II errors [78]. This would allow one to better determine the certainty of meta-analytic results. Unfortunately, it is not recommended for data based on the SMD because it tends to produce naive information sizes [79]. This is problematic for CRF meta-analyses as well as many other meta-analyses when the metric of choice, appropriately, is the SMD. If used, the focus would probably have to be on the original metric, similar to the approach used in the Fong et al., study included in the current review [17].

Fifth, given the large number of systematic reviews with meta-analysis included in the current review [10–13, 16–19, 24–26, 31, 33–36], additional meta-analytic work on the effects of exercise on CRF in adults may be questioned [80]. To assist one in making such a decision, consensus guidelines, including a checklist, have recently been proposed [38]. Along those lines, there may be a need for an updated and more inclusive systematic review with meta-analysis that includes all cancer types so that an examination of the effects of exercise on CRF according to cancer type can be examined.

#### Implications for randomized controlled trials

The results of this review also have several inferences for researchers conducting original randomized controlled trials on exercise and CRF. These suggestions derive from the apparent gaps identified from the included reviews [10–13, 16–19, 24–26, 31, 33–36]. First, for the included reviews that were not limited to cancer type [10, 17–19, 24, 26, 31, 35], a paucity of randomized controlled trials appear to exist for cancers such as prostate, lymphoma, colorectal and leukemia. Assuming this is the case, additional randomized controlled trials focused on these participants may be warranted. Second, since none of the included reviews provided data on the costs associated with the exercise interventions, and assuming that the randomized controlled trials included in these reviews did not collect and report such information [10–13, 16–19, 24–26, 31, 33–36], future randomized controlled trials should collect and report data on cost of the intervention. This is important to others when making decisions regarding what treatment options to recommend and support for improving CRF in adults. Third, based on the lack of data reported in the included reviews as well as reported study quality/risk of bias scores [10–13, 16–19, 24–26, 31, 33–36], it appears that a better job could be done in the collection and/or reporting of data in future randomized controlled trials. This includes such things as (1) the complete reporting of adverse events, (2) methods used for allocation concealment, (3) use of intention-to-treat analyses, (4) compliance to the exercise intervention and (5) data on dropouts, including reasons for dropping out, by group. Fourth, many of the randomized controlled trials included in these previous systematic reviews with meta-analyses appeared to focus on CRF as a secondary versus primary outcome [10–13, 16–19, 24–26, 31, 33–36]. As a result, participants with higher baseline values of CRF may have been excluded from the intervention, thereby diluting the effects of exercise on CRF in those who may benefit most. Fifth, there appeared to be a lack adherence to a theoretical model of behavior change to guide the exercise interventions. This may be important as one included meta-analysis found that exercise interventions that adhered to a theoretical model of behavior change or adaptation model resulted in greater reductions in CRF when compared to those that did not provide such information [10]. However, another found no such association [12]. Finally, future randomized controlled exercise intervention trials may want to consider adhering to the recently developed Consensus on Exercise Reporting Template (CERT) when planning, conducting and reporting randomized controlled exercise intervention trials that assess CRF in adults [81].

### Implications for practice

While the results of the current review regarding improvements in CRF as a result of exercise are inconclusive, exercise does not appear to increase CRF. Given the numerous other potential benefits of exercise in cancer patients [82–85], it would appear plausible to suggest adherence to the NCCN Clinical Practice Guidelines in Oncology guidelines for physical activity as a nonpharmacologic strategy for the management of CRF both during and after treatment [6], as well as more inclusive guidelines recently developed [84]. These recommendations include a goal of 150 min per week of moderate-intensity [3 to 6 metabolic equivalents (METS)] aerobic exercise 3 to 5 days per week as well as resistance training 2 to 3 days per week for 2 sets of 8 to 10 repetitions involving 8 to 10 major muscle groups [84]. All exercise sessions should include a warm-up and a cool down period [84]. Prior to initiating an exercise program, participants should be screened for any effects of disease, treatments and comorbidities [84]. To improve adherence and benefits, it is also recommended that cancer participants exercise in a group or supervised setting [84]. Similar recommendations have also been advocated by others [85].

### Implications for policy

The results of the current study suggest caution regarding any policies including exercise-induced improvements in CRF as justification for promoting exercise in those with cancer. In contrast, policies including the promotion of exercise in cancer patients may be justified by noting that exercise does not appear to increase CRF in adults and while also noting the numerous other benefits that may be gained from participation in such [82–85]. Along those lines, previous evidence-based position statements, systematic reviews with meta-analysis, and systematic reviews of previous systematic reviews with meta-analysis similar to the current study should be helpful to policy-makers [82–85]. However, while evidence should be at the hub of policy-making, numerous other factors need to be considered [86].

Broadly, developing a working relationship with policy-makers and other stakeholders is critical [86]. This is especially true given that policy-makers often come from different backgrounds [86]. Thus, developing a communal language among participants is critical [86]. Finally, similar to obesity policy-making efforts, a combination of minimal but effective policies that safeguard an individual’s independence while at the same time engaging stakeholders in a collaborative way at both the public and private level may be the best approach for successful policy development [87, 88].

### Strengths of current study

From the investigators’ perspective, there are at least four strengths to this study. First, to the best of the authors’ knowledge, this is the first systematic review of previous systematic reviews with meta-analysis regarding the effects of exercise on CRF in adults. As previously delineated, this provides important information for research, practice and policy. Second, 95% PI not reported in any of the original meta-analyses were calculated, thus providing one with what has been suggested to be more accurate information regarding the true effects of exercise on CRF [69]. Third, the NNT was calculated, something that was not done in any of the meta-analyses included in the current review [10–13, 16–19, 24–26, 31, 33–36]. This is important because it provides one with practical information regarding how many people need to exercise in order for one person to see improvements in CRF [10–13, 16–19, 24–26, 31, 33–36]. Fourth, percentile improvements absent from the original meta-analyses were calculated. Since the SMD can often be difficult for others to interpret, percentile improvements provide a more understandable approach regarding the magnitude of effect that exercise has on CRF.

### Potential limitations of current study

In addition to the strengths of the current study, there are several potential limitations. First, while PI are intended to capture the true effects of an intervention on an outcome [69], recent research by Partlett et al. [89], found that PI were only valid if heterogeneity was large, defined as an *I* ^*2*^ value greater than 30%, and study sizes were similar [89]. Consequently, they suggested caution in using 95% PI after conducting a frequentist random-effects meta-analysis such as those conducted in the meta-analyses included in the current review [89]. Second, like any study of this nature, the evidence is not only dependent on the quality of the original meta-analyses but also on the quality of the original studies included in the meta-analysis. Third, given that all included meta-analyses in the current review were based on aggregate data [10–13, 16–19, 24–26, 31, 33–36], the potential for ecological fallacy exists. Fourth, and as previously mentioned, some of the included meta-analyses may have been underpowered to find a true and meaningful effect of exercise on CRF. For example, a post-hoc analysis of Cochrane meta-analyses found that only 22% of the included studies demonstrated at least 80% power to detect a relative risk reduction of 30% [90]. Fifth, different scales consisting of different components and scoring methods were used to assess CRF, thereby potentially confounding overall findings. For example, the two most commonly reported instruments used were the Functional Assessment of Cancer Therapy [10, 11, 13, 16, 17, 19, 24–26, 31, 33, 36] and Piper Fatigue scales [10, 11, 16, 17, 19, 24–26, 31, 35, 36]. The Functional Assessment of Cancer Therapy Scales consist of more than 90 different instruments, categorized according to general measures, cancer-specific measures, cancer specific indices, treatment specific measures, symptom specific measures, non-cancer specific measures and pediatric measures, details of which have been provided elsewhere [91]. These questionnaires include specific instruments for fatigue, administered via self-report or by a trained interviewer either in-person or via telephone [91]. The exact scoring of these instruments vary according to the instrument used [91]. For example, The Functional Assessment of Chronic Illness Therapy Fatigue Scale, a 13-item questionnaire with high internal validity and test-retest reliability assesses a participant’s individual level of fatigue during their usual daily activities over the past week is measured on a four-point Likert scale ranging from 4 (not at all fatigued) to 0 (very much fatigued). In contrast, the Piper Fatigue Scale is a self-report instrument that originally included 42 items and was then revised to include 22 items consisting of four subscales aimed at assessing multidimensional fatigue [92]. Items are scored on a Likert scale ranging from 0 to 10 with higher scores reflective of higher fatigue levels [92]. While inexpensive and easy to administer, all self-report instruments suffer from the well-established potential for social desirability and recall biases.

## Conclusions

Given the lack of certainty regarding the benefits of exercise on CRF in adults, additional well-designed randomized controlled trials and meta-analyses appear warranted. Since exercise does not appear to increase CRF in adults and there are numerous other health benefits that can been derived from such in both cancer patients and survivors, it would appear plausible to suggest that exercise programs that take into consideration the unique needs of cancer patients be recommended.

## Additional files

Additional file 1: Search strategies used for each database. This file includes the search strategies used for all of our electronic databases searches. These include PubMed, Sport Discus, Web of Science, Scopus, Cochrane, ProQuest Dissertations and Theses. (DOCX 224 kb)

Additional file 2: Studies excluded, including reasons for exclusion. This file includes a list of all excluded studies, including the specific reasons for their exclusion. (DOCX 63 kb)

Additional file 3: Item by item results using the AMSTAR assessment instrument. This file includes the results of the AMSTAR assessment for each item from each study. (DOCX 50 kb)

Additional file 4: Post-treatment standardized mean difference (SMD) effect sizes for CRF from included meta-analyses. This file includes the overall post-treatment changes in CRF from included meta-analyses. (DOCX 57 kb)

Additional file 5: NNT and percentile improvements in CRF. This file includes data on NNT and percentile improvements for statistically significant changes in CRF. (DOCX 48 kb)

## Abbreviations

AMSTAR: Assessment of Multiple Systematic Reviews
CI: Confidence intervals
CRF: Cancer-related fatigue
*I*^*2*^: I-squared
NNR: Number needed-to-read
NNT: Number needed-to-treat
PI: Prediction intervals
SMD: Standardized mean difference
