# Supplementary material for: Exercise and cancer-related fatigue in adults: a systematic review of previous systematic reviews with meta-analyses
Source: BMC Cancer. 2017 Oct 23;17:693. doi: 10.1186/s12885-017-3687-5 (PMC5651567; doi:10.1186/s12885-017-3687-5)
Supplement: Supplementary file 1 — Search strategies used for each database. This file includes the search strategies used for all of our electronic databases searches. These include PubMed, Sport Discus, Web of Science, Scopus, Cochrane, ProQuest Dissertations and Theses. (DOCX 224 kb) [file 12885_2017_3687_MOESM1_ESM.docx]

**Additional file 1. Search strategies for electronic database searches.**

**PubMed**

**SportDiscus**

**Web of Science**

**Scopus**

**Cochrane**

**ProQuest Dissertations and Theses**
