# Supplementary material for: Exercise and cancer-related fatigue in adults: a systematic review of previous systematic reviews with meta-analyses
Source: BMC Cancer. 2017 Oct 23;17:693. doi: 10.1186/s12885-017-3687-5 (PMC5651567; doi:10.1186/s12885-017-3687-5)
Supplement: Supplementary file 2 — Studies excluded, including reasons for exclusion. This file includes a list of all excluded studies, including the specific reasons for their exclusion. (DOCX 63 kb) [file 12885_2017_3687_MOESM2_ESM.docx]

**Additional file 2. Studies excluded, including reasons.**

1. Exercise helps reduce cancer-related fatigue. *IDEA Fitness Journal.* 2008;5(7):16-16. Inappropriate Study Design

2. 2013 SYR Accepted Poster Abstracts. *International Journal of Yoga Therapy.* 2013;23 Suppl:32-53. Inappropriate Study Design

3. Adams VM, B.; Baines, S.; Lazarus, C.; Callister, R. A systematic review and meta-analysis of measurements of tongue and hand strength and endurance using the Iowa Oral Performance Instrument (IOPI). *Dysphagia.* 2013;28(3):350-369. Inappropriate Intervention

4. Ahlberg KE, T.; Gaston-Johansson, F.; Mock, V. Assessment and management of cancer-related fatigue in adults. *Lancet.* 2003;362(9384):640-650. Inappropriate Study Design

5. Ahmadi HD, S. Androgen deprivation therapy: evidence-based management of side effects. *Bju International.* 2013;111(4):543-548. Inappropriate Intervention

6. Ahmadi HD, S. Androgen deprivation therapy for prostate cancer: long-term safety and patient outcomes. *Patient Related Outcomes and Measures.* 2014;5:63-70. Inappropriate Intervention

7. Al-Azri MA-A, H.; Al-Moundhri, M. Coping with a diagnosis of breast cancer-literature review and implications for developing countries. *Breast Journal.* 2009;15(6):615-622. Inappropriate Study Design

8. Al-Majid SM, D.O. Cancer-induced fatigue and skeletal muscle wasting: the role of exercise. *Biological Research for Nursing.* 2001;2(3):186-197. Inappropriate Study Design

9. Al-Majid SG, D.P. A biobehavioral model for the study of exercise interventions in cancer-related fatigue. *Biological Research for Nursing.* 2009;10(4):381-391. Inappropriate Study Design

10. Albrecht TAT, A.G. Physical activity in patients with advanced-stage cancer: a systematic review of the literature. *Clinical Journal of Oncology Nursing.* 2012;16(3):293-300. Inappropriate Study Design

11. Alcantara-Silva TRDF, R.; Freitas, N.M.A.; Machado, G.D.P. Fatigue related to radiotherapy for breast and/or gynaecological cancer: a systematic review. *Journal of Clinical Nursing.* 2013;22(19-20):2679-2686. Inappropriate Study Design

12. Aoi WS, K. Does regulation of skeletal muscle function involve circulating microRNAs? *Frontiers in Physiology.* 2014;5:39. Inappropriate Study Design

13. Arden-Close EP, A.; Eiser, C. Health-related quality of life in survivors of lymphoma: a systematic review and methodological critique. *Leukemia & Lymphoma.* 2010;51(4):628-640. Inappropriate Outcomes

14. Armstrong TSS, M.Y.; Breton, G.; Gilbert, M.R.; Mahajan, A.; Scheurer, M.E.; Vera, E.; Berger, A.M. Sleep-wake disturbance in patients with brain tumors. *Neuro-Oncology.* 2016. Inappropriate Study Design

15. Arnold MT, N.F. Does exercise reduce cancer-related fatigue in hospitalised oncology patients? A systematic review. *Onkologie.* 2010;33(11):625-630. Inappropriate Intervention

16. Astroth KSR, C.L.; Welch, J.L. Non-pharmaceutical fatigue interventions in adults receiving hemodialysis: A systematic review. *Nephrology Nursing Journal.* 2013;40(5):407-427. Inappropriate Population

17. Balsamo SD, L.R.; dos Santos-Neto, L.L.; da Mota, L.M. Exercise and fatigue in rheumatoid arthritis. *Israel Medical Association Journal.* 2014;16(1):57-60. Inappropriate Population

18. Barker-Collo SF, V.L.; Dudley, M. Post stroke fatigue--where is the evidence to guide practice? *New Zealand Medical Journal.* 2007;120(1264):U2780. Inappropriate Population

19. Barsevick AMN, T.; Brown, S. Management of cancer-related fatigue. *Clinical Journal of Oncology Nursing.* 2008;12(5 Suppl):21-25. Inappropriate Study Design

20. Barsevick AMI, M.R.; Hinds, P.; Miller, A.; Berger, A.; Jacobsen, P.; Ancoli-Israel, S.; Reeve, B.B.; Mustian, K.; O'Mara, A.; Lai, J.S.; Fisch, M.; Cella, D. Recommendations for high-priority research on cancer-related fatigue in children and adults. *Journal of the National Cancer Institute.* 2013;105(19):1432-1440. Inappropriate Study Design

21. Battaglini CL. Physical activity and hematological cancer survivorship. *Recent Results in Cancer Research.* 2011;186:275-304. Inappropriate Study Design

22. Battaglini CLM, R.C.; Phillips, B.L.; Lee, J.T.; Story, C.E.; Nascimento, M.G.; Hackney, A.C. Twenty-five years of research on the effects of exercise training in breast cancer survivors: A systematic review of the literature. *World Journal of Clinical Oncology.* 2014;5(2):177-190. Inappropriate Study Design

23. Baumann FTZ, E.M.; Bloch, W. Clinical exercise interventions in prostate cancer patients--a systematic review of randomized controlled trials. *Supportive Care in Cancer.* 2012;20(2):221-233. Inappropriate Study Design

24. Baumann FTB, W.; Weissen, A.; Brockhaus, M.; Beulertz, J.; Zimmer, P.; Streckmann, F.; Zopf, E.M. Physical activity in breast cancer patients during medical treatment and in the aftercare - a review. *Breast Care.* 2013;8(5):330-334. Inappropriate Study Design

25. Baumann FTB, W.; Beulertz, J. Clinical exercise interventions in pediatric oncology: a systematic review. *Pediatric Research.* 2013;74(4):366-374. Inappropriate Population

26. Beaton RP-F, W.; Robertson, C.; Vigar, C.; Watson, H.; Harris, S.R. Effects of exercise intervention on persons with metastatic cancer: A systematic review. *Physiotherapy Canada.* 2009;61(3):141-153. Inappropriate Study Design

27. Benzo RP. Pulmonary rehabilitation in lung cancer: a scientific opportunity. *Journal of Cardiopulmonary Rehabilitation and Prevention.* 2007;27(2):61-64. Inappropriate Study Design

28. Bergenthal NW, A.; Streckmann, F.; Wolkewitz, K.D.; Monsef, I.; Engert, A.; Elter, T.; Skoetz, N. Aerobic physical exercise for adult patients with haematological malignancies. *Cochrane Database of Systematic Reviews.* 2014(11). Inappropriate Outcomes

29. Berger AMG, L.H.; Mayer, D.K. Cancer-related fatigue: implications for breast cancer survivors. *Cancer.* 2012;118(8 Suppl):2261-2269. Inappropriate Study Design

30. Berger AMY, S.; Million, R. Update on interventions focused on symptom clusters: what has been tried and what have we learned? *Current Opinion in Supportive Palliative Care.* 2013;7(1):60-66. Inappropriate Study Design

31. Binkley JMH, S.R.; Levangie, P.K.; Pearl, M.; Guglielmino, J.; Kraus, V.; Rowden, D. Patient perspectives on breast cancer treatment side effects and the prospective surveillance model for physical rehabilitation for women with breast cancer. *Cancer.* 2012;118(8 Suppl):2207-2216. Inappropriate Study Design

32. Bird MLC, M.J.; Williams, A.D. Accidental fall rates in community-dwelling adults compared to cancer survivors during and post-treatment: A systematic review with meta-analysis. *Oncology Nursing Forum.* 2016;43(2):E64-E72. Inappropriate Study Design

33. Bourke LS, D.; Steed, L.; Hooper, R.; Carter, A.; Catto, J.; Albertsen, P.C.; Tombal, B.; Payne, H.A.; Rosario, D.J. Exercise for men with prostate cancer: A systematic review and meta-analysis. *European Urology.* 2016;69(4):693-703. Inappropriate Outcomes

34. Braam KIvdT, P.; Takken, T.; Veening, M.A.; van Dulmen-den, Broeder E.; Kaspers, G.J. Physical exercise training interventions for children and young adults during and after treatment for childhood cancer. *Cochrane Database of Systematic Reviews.* 2013(4):CD008796. Inappropriate Population

35. Braam KIvdT, P.; Takken, T.; Veening, M.A.; van Dulmen-den, Broeder E.; Kaspers, G.J. Physical exercise training interventions for children and young adults during and after treatment for childhood cancer. *Cochrane Database of Systematic Reviews.* 2016;3:CD008796. Inappropriate Population

36. Bradt JG, S.W.; Dileo, C. Dance/movement therapy for improving psychological and physical outcomes in cancer patients. *Cochrane Database of Systematic Reviews.* 2011(10):CD007103. Inappropriate Study Design

37. Brown JCW-S, K.; Lee, A.; Schmitz, K.H. Cancer, physical activity, and exercise. *Comprehensive Physiology.* 2012;2(4):2775-2809. Inappropriate Study Design

38. Buffart LMvU, J.G.; Riphagen, I.I.; Brug, J.; van, Mechelen W.; Brown, W.J.; Chinapaw, M.J. Physical and psychosocial benefits of yoga in cancer patients and survivors, a systematic review and meta-analysis of randomized controlled trials. *BMC Cancer.* 2012;12:559. Inappropriate Intervention

39. Campbell KLN, S.E.; Winters-Stone, K.M. Review of exercise studies in breast cancer survivors: attention to principles of exercise training. *British Journal of Sports Medicine.* 2012;46(13):909-916. Inappropriate Outcomes

40. Campos MPH, B.J.; Riechelmann, R.; Del, Giglio A. Cancer-related fatigue: a review. *Revista da Associacao Medica Brasileira.* 2011;57(2):211-219. Inappropriate Study Design

41. Capozzi LCN, K.C.; McNeely, M.L.; Lau, H.; Culos-Reed, S.N. The impact of physical activity on health-related fitness and quality of life for patients with head and neck cancer: a systematic review. *British Journal of Sports Medicine.* 2016;50(6):325-338. Inappropriate Study Design

42. Carson JLT, M.L.; Jay, M. Anemia and postoperative rehabilitation. *Canadian Journal of Anesthesia.* 2003;50(6 Suppl):S60-S64. Inappropriate Study Design

43. Cassileth BR. Psychiatric benefits of integrative therapies in patients with cancer. *International Review of Psychiatry.* 2014;26(1):114-127. Inappropriate Study Design

44. Cavalheri VT, F.; Nonoyama, M.; Jenkins, S.; Hill, K. Exercise training undertaken by people within 12 months of lung resection for non-small cell lung cancer. *Cochrane Database of Systematic Reviews.* 2013(7). Inappropriate Outcomes

45. Chang CWM, P.F.; Jou, S.T.; Wong, T.T.; Chen, Y.C. Systematic review and meta-analysis of nonpharmacological interventions for fatigue in children and adolescents with cancer. *Worldviews on Evidence-Based Nursing.* 2013;10(4):208-217. Inappropriate Population

46. Chiu HYH, H.C.; Chen, P.Y.; Hou, W.H.; Tsai, P.S. Walking improves sleep in individuals with cancer: A meta-analysis of randomized, controlled trials. *Oncology Nursing Forum.* 2015;42(2):E54-E62. Inappropriate Outcomes

47. Cigna JA. Home care physical therapy for the cancer patient. *Home Healthcare Nurse.* 2007;25(3):158-161. Inappropriate Study Design

48. Conn VSH, A.R.; Porock, D.C.; McDaniel, R.; Nielsen, P.J. A meta-analysis of exercise interventions among people treated for cancer. *Supportive Care in Cancer.* 2006;14(7):699-712. Inappropriate Intervention

49. Cormie PN, R.U.; Taaffe, D.R.; Spry, N.; Galvao, D.A. Exercise therapy for sexual dysfunction after prostate cancer. *Nature Reviews Urology.* 2013;10(12):731-736. Inappropriate Study Design

50. Cormie PN, A.K.; Chambers, S.K.; Galvao, D.A.; Newton, R.U. The potential role of exercise in neuro-oncology. *Frontiers in Oncology.* 2015;5:85. Inappropriate Study Design

51. Coughlin SSY, W.; Whitehead, M.S.; Smith, S.A. Advancing breast cancer survivorship among African-American women. *Breast Cancer Research and Treatment.* 2015;153(2):253-261. Inappropriate Study Design

52. Courneya KSF, C.M. Physical exercise and quality of life following cancer diagnosis: a literature review. *Annals of Behavioral Medicine.* 1999;21(2):171-179. Inappropriate Study Design

53. Coyne JCT, B.D.; Hagedoorn, M. Ain't necessarily so: Review and critique of recent meta-analyses of behavioral medicine interventions in health psychology. *Health Psychology.* 2010;29(2):107-116. Inappropriate Study Design

54. Craft LLV, E.H.; Helenowski, I.B.; Rademaker, A.W.; Courneya, K.S. Exercise effects on depressive symptoms in cancer survivors: a systematic review and meta-analysis. *Cancer Epidemiology, Biomarkers & Prevention.* 2012;21(1):3-19. Inappropriate Outcomes

55. Cramer HH, H.; Dobos, G.; Lauche, R. A systematic review and meta-analysis estimating the expected dropout rates in randomized controlled trials on yoga interventions. *Evidence-Based Complementary and Alternative Medicine.* 2016. Inappropriate Intervention

56. Cramp FD, J. Exercise for the management of cancer-related fatigue in adults. *Cochrane Database of Systematic Reviews.* 2008(2). Inappropriate Intervention

57. Cramp FB-D, J. Exercise for the management of cancer-related fatigue in adults. *Cochrane Database of Systematic Reviews.* 2012(11). Inappropriate Intervention

58. Crandall KM, R.; Campbell, A.; Kearney, N. Exercise intervention for patients surgically treated for Non-Small Cell Lung Cancer (NSCLC): a systematic review. *Surgical Oncology.* 2014;23(1):17-30. Inappropriate Study Design

59. Crevenna RZ, C.; Keilani, M.Y.; Schmidinger, M.; Bittner, C.; Nuhr, M.; Nur, H.; Marosi, C.; Fialka-Moser, V.; Quittan, M. [Aerobic endurance training for cancer patients]. *Wiener medizinische Wochenschrift.* 2003;153(9-10):212-216. Inappropriate Study Design

60. Davis MPW, D. Mechanisms of fatigue. *Journal of Supportive Oncology.* 2010;8(4):164-174. Inappropriate Study Design

61. Davis MPG, H.W. Long-term and short-term effects of insomnia in cancer and effective interventions. *Cancer Journal.* 2014;20(5):330-344. Inappropriate Study Design

62. Day JG, D.C.; Rooney, A.G.; Bulbeck, H.J.; Zienius, K.; Boele, F.; Grant, R. Neurocognitive deficits and neurocognitive rehabilitation in adult brain tumors. *Current Treatment Options in Neurology.* 2016;18(5):22. Inappropriate Study Design

63. De Backer ICS, G.; Backx, F.J.; Vreugdenhil, G.; Kuipers, H. Resistance training in cancer survivors: A systematic review. *International Journal of Sports Medicine.* 2009;30(10):703-712. Inappropriate Intervention

64. De WSV, Belle S. Cancer-related fatigue. *Acta Clinica Belgica.* 2010;65(6):378-385. Inappropriate Study Design

65. del Pino-Sedeno TT-M, M.M.; Ruiz-Irastorza, G.; Cuellar-Pompa, L.; de Pascual-Medina, A.M.; Serrano-Aguilar, P. Effectiveness of nonpharmacologic interventions for decreasing fatigue in adults with systemic lupus erythematosus: A systematic review. *Arthritis Care & Research.* 2016;68(1):141-148. Inappropriate Population

66. Demark-Wahnefried WC, K.L.; Hayes, S.C. Weight management and its role in breast cancer rehabilitation. *Cancer.* 2012;118(8 Suppl):2277-2287. Inappropriate Study Design

67. Dennett AMP, C.L.; Shields, N.; Prendergast, L.A.; Taylor, N.F. Moderate-intensity exercise reduces fatigue and improves mobility in cancer survivors: a systematic review and meta-regression. *Journal of Physiotherapy.* 2016;62(2):68-82. Inappropriate Intervention

68. Dieli-Conwright CMO, B.Z. Exercise after breast cancer treatment: current perspectives. *Breast Cancer.* 2015;7:353-362. Inappropriate Study Design

69. Dimeo F. Radiotherapy-related fatigue and exercise for cancer patients: a review of the literature and suggestions for future research. *Frontiers of Radiation Therapy and Oncology.* 2002;37:49-56. Inappropriate Study Design

70. Dimeo FC. Effects of exercise on cancer-related fatigue. *Cancer.* 2001;92(6 Suppl):1689-1693. Inappropriate Study Design

71. Dirks AJJ, K.M. Statin-induced apoptosis and skeletal myopathy. *American Journal of Physiology - Cell Physiology.* 2006;291(6):C1208-C1212. Inappropriate Outcomes

72. DiStasio SA. Integrating yoga into cancer care. *Clinical Journal of Oncology Nursing.* 2008;12(1):125-130. Inappropriate Intervention

73. Du SZH, L.L.; Dong, J.S.; Xu, G.H.; Jin, S.J.; Zhang, H.; Yin, H.Y. Patient education programs for cancer-related fatigue: A systematic review. *Patient Education and Counseling.* 2015;98(11):1308-1319. Inappropriate Study Design

74. Durstine JLP, P.; Franklin, B.A.; Morgan, D.; Pitetti, K.H.; Roberts, S.O. Physical activity for the chronically ill and disabled. *Sports Medicine.* 2000;30(3):207-219. Inappropriate Study Design

75. Eckersley RM. Losing the battle of the bulge: causes and consequences of increasing obesity. *Medical Journal of Australia.* 2001;174(11):590-592. Inappropriate Study Design

76. Egan MYM, S.; Sikora, L.; Chasen, M.; Fitch, M.; Eldred, S. Rehabilitation following cancer treatment. *Disability and Rehabilitation.* 2013;35(26):2245-2258. Inappropriate Study Design

77. Eickmeyer SMG, G.L.; Shahpar, S.; Do, K.D. The role and efficacy of exercise in persons with cancer. *Physical Medicine and Rehabilitation.* 2012;4(11):874-881. Inappropriate Study Design

78. Eliakim AN, D. Endogenous hyperandrogenism and exercise capacity lessons from the exercise-congenital adrenal hyperplasia model. *Journal of Pediatric Endocrinology and Metabolism.* 2010;23(12):1213-1219. Inappropriate Intervention

79. Elliott SL, D.M.; Walker, L.M.; Wassersug, R.; Robinson, J.W. Androgen deprivation therapy for prostate cancer: recommendations to improve patient and partner quality of life. *Journal of Sexual Medicine.* 2010;7(9):2996-3010. Inappropriate Intervention

80. Escalante CP. Treatment of cancer-related fatigue: an update. *Supportive Care in Cancer.* 2003;11(2):79-83. Inappropriate Study Design

81. Escalante CPM, E.F. Cancer-related fatigue: the approach and treatment. *Journal of General Internal Medicine.* 2009;24 Suppl 2:S412-S416. Inappropriate Study Design

82. Eva GW, B. Rehabilitation in end-of-life management. *Current Opinion in Supportive Palliative Care.* 2010;4(3):158-162. Inappropriate Outcomes

83. Evans MB, S.; Huntley, A.L.; Feder, G. Cancer patients' experiences of using mistletoe (Viscum album): A qualitative systematic review and synthesis. *Journal of Alternative and Complementary Medicine.* 2016;22(2):134-144. Inappropriate Intervention

84. Evans WJ. Physical function in men and women with cancer. Effects of anemia and conditioning. *Oncology.* 2002;16(9 Suppl 10):109-115. Inappropriate Study Design

85. Evans WJL, C.P. Physiological basis of fatigue. *American Journal of Physical Medicine & Rehabilitation.* 2007;86(1 Suppl):S29-S46. Inappropriate Study Design

86. Eyigor SK, S. Exercise in patients coping with breast cancer: An overview. *World Journal of Clinical Oncology.* 2014;5(3):406-411. Inappropriate Study Design

87. Felbel SM, Joerg J.; Monsef, Ina; Engert, Andreas; Skoetz, Nicole. Yoga in addition to standard care for patients with haematological malignancies. *Cochrane Database of Systematic Reviews.* 2014. Inappropriate Intervention

88. Fialka-Moser VC, R.; Korpan, M.; Quittan, M. Cancer rehabilitation: particularly with aspects on physical impairments. *Journal of Rehabilitation Medicine.* 2003;35(4):153-162. Inappropriate Study Design

89. Finaud JL, G.; Filaire, E. Oxidative stress : relationship with exercise and training. *Sports Medicine.* 2006;36(4):327-358. Inappropriate Outcomes

90. Fiuza-Luces CG, N.; Simpson, R.J.; Berger, N.A.; Ramirez, M.; Lucia, A. Understanding graft-versus-host disease. Preliminary findings regarding the effects of exercise in affected patients. *Exercise Immunology Review.* 2015;21:80-112. Inappropriate Study Design

91. Fleishman SB. Treatment of symptom clusters: pain, depression, and fatigue. *Journal of the National Cancer Institute Monographs.* 2004(32):119-123. Inappropriate Study Design

92. Focht BCC, S.K.; Devor, S.T.; Garver, M.J.; Lucas, A.R.; Thomas-Ahner, J.M.; Grainger, E. Resistance exercise interventions during and following cancer treatment: a systematic review. *Journal of Supportive Oncology.* 2013;11(2):45-60. Inappropriate Study Design

93. Fontein DBYdG, N.A.; Duijm, M.; Bastiaannet, E.; Portielje, J.E.A.; Van de Velde, C.J.H.; Liefers, G.J. Age and the effect of physical activity on breast cancer survival: A systematic review. *Cancer Treatment Reviews.* 2013;39(8):958-965. Inappropriate Study Design

94. Foss K. Inflammation and cancer-related fatigue in breast cancer survivors. *ProQuest Dissertations and Theses.* 2013:36. Inappropriate Study Design

95. Friendenreich CMC, K.S. Exercise as rehabilitation for cancer patients. *Clinical Journal of Sport Medicine.* 1996;6(4):237-244. Inappropriate Study Design

96. Furmaniak ACM, M.; Markes, M.H. Effect of exercise on fatigue and depression in women undergoing adjuvant treatment for breast cancer: A meta-analysis. *Journal of Psychosomatic Research.* 2016;85:65-66. Inappropriate Study Design

97. Garcia DOT, C.A. Physical activity and cancer survivorship. *Nutrition in Clinical Practice.* 2014;29(6):768-779. Inappropriate Study Design

98. Gardner JRL, P.M.; Fraser, S.F. Effects of exercise on treatment-related adverse effects for patients with prostate cancer receiving androgen-deprivation therapy: a systematic review. *Journal of Clinical Oncology.* 2014;32(4):335-346. Inappropriate Study Design

99. Gerritsen JKWV, A.J.P.E. Exercise improves quality of life in patients with cancer: a systematic review and meta-analysis of randomised controlled trials. *British Journal of Sports Medicine.* 2016;50(13):796-803. Inappropriate Intervention

100. Gilliam LASC, D.K. Chemotherapy-induced weakness and fatigue in skeletal muscle: the role of oxidative stress. *Antioxidants & Redox Signaling.* 2011;15(9):2543-2563. Inappropriate Intervention

101. Gilligan T. Quality of life among testis cancer survivors. *Urologic Oncology.* 2015;33(9):413-419. Inappropriate Study Design

102. Giuliani AC, B. Exercise, free radical generation and vitamins. *European Journal of Cancer Prevention.* 1997;6 Suppl 1:S55-S67. Inappropriate Study Design

103. Goedendorp MMG, M.F.M.; Verhagen, C.A.H.H.; Bleijenberg, G. Psychosocial interventions for reducing fatigue during cancer treatment in adults. *Cochrane Database of Systematic Reviews.* 2009(1). Inappropriate Intervention

104. Grabenbauer AG, A.J.; Lengenfelder, R.; Grabenbauer, G.G.; Distel, L.V. Feasibility of a 12-month-exercise intervention during and after radiation and chemotherapy in cancer patients: impact on quality of life, peak oxygen consumption, and body composition. *Radiation Oncology.* 2016;11. Inappropriate Study Design

105. Grande AJS, V.; Maddocks, M. Exercise for cancer cachexia in adults: Executive summary of a Cochrane Collaboration systematic review. *Journal of Cachexia Sarcopenia and Muscle.* 2015;6(3):208-211. Inappropriate Study Design

106. Grande AJS, Valter; Riera, Rachel; Medeiros, Alessandra; Vitoriano-Simone, G.P.; Peccin, Maria S.; Maddocks, Matthew. Exercise for cancer cachexia in adults. *Cochrane Database of Systematic Reviews.* 2014. Inappropriate Study Design

107. Granger CLM, C.F.; Berney, S.; Chao, C.; Denehy, L. Exercise intervention to improve exercise capacity and health related quality of life for patients with Non-small cell lung cancer: A systematic review. *Lung Cancer.* 2011;72(2):139-153. Inappropriate Study Design

108. Gupta AAP, J.K.; Jones, J.M.; Amin, L.; Chang, E.K.; Korenblum, C.; Mina, D.S.; McCabe, L.; Mitchell, L.; Giuliani, M.E. Reimagining care for adolescent and young adult cancer programs: Moving with the times. *Cancer.* 2016;122(7):1038-1046. Inappropriate Population

109. Hamlin SKV, P.S.; Kanusky, J.T.; Shaw, A.D. Role of diastole in left ventricular function, II: diagnosis and treatment. *American Journal of Critical Care.* 2004;13(6):453-466. Inappropriate Outcomes

110. Harder HP, L.; Jenkins, V. Randomised controlled trials of yoga interventions for women with breast cancer: a systematic literature review. *Supportive Care in Cancer.* 2012;20(12):3055-3064. Inappropriate Intervention

111. Harris JD. Fatigue in chronically ill patients. *Current Opinion in Supportive Palliative Care.* 2008;2(3):180-186. Inappropriate Study Design

112. Hartvig HP. Molecular mechanisms in cytotoxic drug induced fatigue. *Annales Pharmaceutiques Francaises.* 2010;68(2):76-81. Inappropriate Study Design

113. Hasenoehrl TK, M.; Sedghi, Komanadj T.; Mickel, M.; Margreiter, M.; Marhold, M.; Crevenna, R. The effects of resistance exercise on physical performance and health-related quality of life in prostate cancer patients: a systematic review. *Supportive Care in Cancer.* 2015;23(8):2479-2497. Inappropriate Study Design

114. Hewitt JAM, K.; van Someren, K.A.; Jewell, A.P.; Garrod, R. Exercise for breast cancer survival: the effect on cancer risk and cancer-related fatigue (CRF). *International Journal of Fertility and Women's Medicine.* 2005;50(5 Pt 1):231-239. Inappropriate Study Design

115. Holland AEW, K.; Spruit, M.A. How to adapt the pulmonary rehabilitation programme to patients with chronic respiratory disease other than COPD. *European Respiratory Review.* 2013;22(130):577-586. Inappropriate Population

116. Horneber MF, I.; Dimeo, F.; Ruffer, J.U.; Weis, J. Cancer-related fatigue epidemiology, pathogenesis, diagnosis, and treatment. *Deutsches Arzteblatt International.* 2012;109(9):161-U135. Inappropriate Study Design

117. Ingram CV, C. Exercise intervention to modify physiologic risk factors in cancer survivors. *Seminars in Oncology Nursing.* 2007;23(4):275-284. Inappropriate Study Design

118. Innominato PFR, V.P.; Palesh, O.G.; Ulusakarya, A.; Spiegel, D.; Levi, F.A. The circadian timing system in clinical oncology. *Annals of Medicine.* 2014;46(4):191-207. Inappropriate Intervention

119. Irwin MLA, B.E. Physical activity interventions following cancer diagnosis: methodologic challenges to delivery and assessment. *Cancer Investigation.* 2004;22(1):30-50. Inappropriate Study Design

120. Jack SW, M.; Grocott, M.P. Perioperative exercise training in elderly subjects. *Best Practice & Research Clinical Anaesthesiology.* 2011;25(3):461-472. Inappropriate Study Design

121. Jacobsen PBT, C.L. Fatigue in the radiation therapy patient: current management and investigations. *Seminars in Radiation Oncology.* 2003;13(3):372-380. Inappropriate Study Design

122. Jain SB, C.; Fiorentino, L.; Khorsan, R.; Crawford, C. Are there efficacious treatments for treating the fatigue-sleep disturbance-depression symptom cluster in breast cancer patients? A Rapid Evidence Assessment of the Literature (REAL (c)). *Breast Cancer-Targets and Therapy.* 2015;7:267-291. Inappropriate Study Design

123. Jedlicka FE, L.; Vasova, I.; Tomaskova, I.; Vorlicek, J.; Spinar, J. [Chronic fatigue syndrome in cancer patients. Diagnostic and treatment options]. *Vnitrni Lekarstvi.* 2007;53(9):979-985. Inappropriate Study Design

124. Jereczek-Fossa BAM, H.R.; Orecchia, R. Radiotherapy-related fatigue: how to assess and how to treat the symptom. A commentary. *Tumori.* 2001;87(3):147-151. Inappropriate Study Design

125. Jereczek-Fossa BAM, H.R.; Orecchia, R. Radiotherapy-related fatigue. *Critical Reviews in Oncology/Hematology.* 2002;41(3):317-325. Inappropriate Study Design

126. Johnson BTL, R.E.; MacDonald, H.V. Panning for the gold in health research: Incorporating studies' methodological quality in meta-analysis. *Psychology & Health.* 2015;30(1):135-152. Inappropriate Outcomes

127. Jones LWA, C.M. Exercise-oncology research: Past, present, and future. *Acta Oncologica.* 2013;52(2):195-215. Inappropriate Study Design

128. Jones MS, B.; Just, J.; Fallowfield, L. Epoetin alfa improves quality of life in patients with cancer: Results of a metaanalysis. *Cancer.* 2004;101(8):1720-1732. Inappropriate Intervention

129. Jung MZ, N.; Fuchs, B. Exercise therapy for children with cancer related fatigue. *Klinische Padiatrie.* 2016;228(3):157-163. Inappropriate Population

130. Kampshoff CSJ, F.; van, Mechelen W.; May, A.M.; Brug, J.; Chinapaw, M.J.; Buffart, L.M. Determinants of exercise adherence and maintenance among cancer survivors: a systematic review. *International Journal of Behavioral Nutrition and Physical Activity.* 2014;11:80. Inappropriate Outcomes

131. Karki A. Physiotherapy for the functioning of breast cancer patients: Studies of the effectiveness of physiotherapy methods and exercise, of the content and timing of postoperative education and of the experienced functioning and disability. *ProQuest Dissertations and Theses.* 2005:70. Inappropriate Study Design

132. Keogh JWLM, R.D. Body composition, physical fitness, functional performance, quality of life, and fatigue benefits of exercise for prostate cancer patients: A systematic review. *Journal of Pain and Symptom Management.* 2012;43(1):96-110. Inappropriate Study Design

133. Khamoui AVK, J.S. Candidate mechanisms underlying effects of contractile activity on muscle morphology and energetics in cancer cachexia. *European Journal of Cancer Care.* 2012;21(2):143-157. Inappropriate Study Design

134. Kirkham AAB, K.A.; Sayyari, S.; Campbell, K.L.; Davis, M.K. Clinically relevant physical benefits of exercise interventions in breast cancer survivors. *Current Oncology Reports.* 2016;18(2):1-9. Inappropriate Study Design

135. Kirshbaum M. Promoting physical exercise in breast cancer care. *Nursing Standard.* 2005;19(41):41-48. Inappropriate Study Design

136. Kirshbaum MN. A review of the benefits of whole body exercise during and after treatment for breast cancer. *Journal of Clinical Nursing.* 2007;16(1):104-121. Inappropriate Study Design

137. Klein PJS, R.; Rhoads, C.J. Qigong in cancer care: a systematic review and construct analysis of effective Qigong therapy. *Supportive Care in Cancer.* 2016;24(7):3209-3222. Inappropriate Intervention

138. Knobf MTM, R.; Dorward, J. Exercise and quality of life outcomes in patients with cancer. *Seminars in Oncology Nursing.* 2007;23(4):285-296. Inappropriate Study Design

139. Knobf MTC, J. Lifestyle interventions for cardiovascular risk reduction in women with breast cancer. *Current Cardiology Reviews.* 2011;7(4):250-257. Inappropriate Study Design

140. Koutoukidis DAK, M.T.; Lanceley, A. Obesity, diet, physical activity, and health-related quality of life in endometrial cancer survivors. *Nutrition Reviews.* 2015;73(6):399-408. Inappropriate Study Design

141. Kuchinski AMR, M.; Lash, A.A. Treatment-related fatigue and exercise in patients with cancer: a systematic review. *Medsurg Nursing.* 2009;18(3):174-180. Inappropriate Study Design

142. Labourey JL. Physical activity in the management of cancer-related fatigue induced by oncological treatments. *Annales de Readaptation et de Medecine Physique.* 2007;50(6):450-459. Inappropriate Study Design

143. Larkin DL, V.; Aromataris, E. Managing cancer-related fatigue in men with prostate cancer: A systematic review of non-pharmacological interventions. *International Journal of Nursing Practice.* 2014;20(5):549-560. Inappropriate Study Design

144. Lawrence DPK, B.; Miller, K.; Devine, D.; Lau, J. Evidence report on the occurrence, assessment, and treatment of fatigue in cancer patients. *Journal of the National Cancer Institute Monographs.* 2004(32):40-50. Inappropriate Study Design

145. Leak BAL, Walton A.; Shaw-Kokot, J.; Mayer, D.K.; Reeve, B.B. Patient-reported symptoms and quality of life in adults with acute leukemia: a systematic review. *Oncology Nursing Forum.* 2015;42(2):E91-E101. Inappropriate Study Design

146. Lee KA. Sleep and fatigue. *Annual Review of Nursing Research.* 2001;19:249-273. Inappropriate Study Design

147. Lesage PP, R.K. Management of fatigue in the cancer patient. *Oncology.* 2002;16(3):373-378, 381. Inappropriate Study Design

148. Ligibel J. Lifestyle factors in cancer survivorship. *Journal of Clinical Oncology.* 2012;30(30):3697-3704. Inappropriate Study Design

149. Lindheimer JBOC, P.J.; Dishman, R.K. Quantifying the placebo effect in psychological outcomes of exercise training: A meta-analysis of randomized trials. *Sports Medicine.* 2015;45(5):693-711. Inappropriate Population

150. Lipman AJL, D.P. The management of fatigue in cancer patients. *Oncology.* 2004;18(12):1527-1535. Inappropriate Study Design

151. Litterini AJJ, Diane U. Exercise for managing cancer-related fatigue. *Physical Therapy.* 2011;91(3):301-304. Inappropriate Study Design

152. Littlewood TC, G. Epoetin alfa: basic biology and clinical utility in cancer patients. *Expert Review of Anticancer Therapy.* 2005;5(6):947-956. Inappropriate Study Design

153. Liu RDKSC, M.J.M.; Huijgens, P.C.; van Mechelen, W. Physical exercise interventions in haematological cancer patients, feasible to conduct but effectiveness to be established: A systematic literature review. *Cancer Treatment Reviews.* 2009;35(2):185-192. Inappropriate Study Design

154. Lof MB, K.; Weiderpass, E. Physical activity and biomarkers in breast cancer survivors: a systematic review. *Maturitas.* 2012;73(2):134-142. Inappropriate Study Design

155. Lopes-Junior LCB, E.O.; Nascimento, L.C.; Nunes, M.D.; Pereira-da-Silva, G.; Lima, R.A. Non-pharmacological interventions to manage fatigue and psychological stress in children and adolescents with cancer: an integrative review. *European Journal of Cancer Care.* 2015. Inappropriate Population

156. Loprinzi CLW, S.L.; Barton, D.L.; Laack, N.N. Symptom management in premenopausal patients with breast cancer. *Lancet Oncology.* 2008;9(10):993-1001. Inappropriate Intervention

157. Loprinzi PDC, B.J. Effects of physical activity on common side effects of breast cancer treatment. *Breast Cancer.* 2012;19(1):4-10. Inappropriate Study Design

158. Lotfi-Jam KC, M.; Jefford, M.; Schofield, P.; Charleson, C.; Aranda, S. Nonpharmacologic strategies for managing common chemotherapy adverse effects: A systematic review. *Journal of Clinical Oncology.* 2008;26(34):5618-5629. Inappropriate Study Design

159. Loughney LW, M.A.; Kemp, G.J.; Grocott, M.P.W.; Jack, S. Exercise intervention in people with cancer undergoing adjuvant cancer treatment following surgery: A systematic review. European Journal of Surgical Oncology; 2015, 2015. Inappropriate Study Design

160. Loughney LW, M.A.; Kemp, G.J.; Grocott, M.P.W.; Jack, S. Exercise intervention in people with cancer undergoing neoadjuvant cancer treatment and surgery: A systematic review. European Journal of Surgical Oncology; 2016, 2016. Inappropriate Study Design

161. Lowe SS. Physical activity and palliative cancer care. *Recent Results Cancer Res.* 2011;186:349-365. Inappropriate Study Design

162. Lowe SSW, S.M.; Baracos, V.E.; Courneya, K.S. Home-based functional walking program for advanced cancer patients receiving palliative care: a case series. *Bmc Palliative Care.* 2013;12:22. Inappropriate Study Design

163. Lowe SST, M.; Faily, J.; Watanabe, S.M.; Courneya, K.S. Physical activity in advanced cancer patients: a systematic review protocol. *Systematic Reviews.* 2016;5:43. Inappropriate Study Design

164. Luctkar-Flude MFG, D.L.; Tranmer, J.E.; Woodend, K. Fatigue and physical activity in older adults with cancer: a systematic review of the literature. *Cancer Nursing.* 2007;30(5):E35-E45. Inappropriate Study Design

165. Luctkar-Flude M. Fatigue, physical activity, physical functioning and quality of life in older adults with cancer. *ProQuest Dissertations and Theses.* 2007:172. Inappropriate Study Design

166. MacDonald N. Cancer cachexia and targeting chronic inflammation: a unified approach to cancer treatment and palliative/supportive care. *Journal of Supportive Oncology.* 2007;5(4):157-162. Inappropriate Study Design

167. Mackinnon LT. Future directions in exercise and immunology: regulation and integration. *International Journal of Sports Medicine.* 1998;19 Suppl 3:S205-S209. Inappropriate Study Design

168. Maddocks MG, Wei; Higginson, Irene J.; Wilcock, Andrew. Neuromuscular electrical stimulation for muscle weakness in adults with advanced disease. *Cochrane Database of Systematic Reviews.* 2013. Inappropriate Intervention

169. Mansel JKC, E.C. Nonpharmacologic approach to sleep disorders. *Cancer Journal.* 2014;20(5):345-351. Inappropriate Study Design

170. Manzullo EL, W.; Escalante, C. Treatment for cancer-related fatigue: an update. *Expert Review of Anticancer Therapy.* 2003;3(1):99-106. Inappropriate Study Design

171. Manzullo EFE, C.P. Research into fatigue. *Hematology/Oncology Clinics of North America.* 2002;16(3):619-628. Inappropriate Study Design

172. Markes MB, T.; Resch, K.L. Exercise for women receiving adjuvant therapy for breast cancer. *Cochrane Database of Systematic Reviews.* 2006(4):CD005001. Inappropriate Study Design

173. Markes M. Exercise as an intervention during breast cancer treatment. *Exercise and Cancer Survivorship: Impact on Health Outcomes and Quality of Life.* 2010:37-51. Inappropriate Study Design

174. Maughan DT, M. Discerning primary and secondary factors responsible for clinical fatigue in multisystem diseases. *Biology (Basel).* 2014;3(3):606-622. Inappropriate Study Design

175. McClellan R. Exercise programs for patients with cancer improve physical functioning and quality of life. *Journal of Physiotherapy.* 2013;59(1):57. Inappropriate Study Design

176. McMillan E. Using exercise based interventions for the treatment of cancer related fatigue: A meta analysis. *ProQuest Dissertations and Theses.* 2009:203. Inappropriate Study Design

177. McMillan EMN, Ian J. Exercise is an effective treatment modality for reducing cancer-related fatigue and improving physical capacity in cancer patients and survivors: a meta-analysis. *Applied Physiology, Nutrition & Metabolism.* 2011;36(6):892-903. Inappropriate Study Design

178. McNeely MLC, K.L.; Rowe, B.H.; Klassen, T.P.; Mackey, J.R.; Courneya, K.S. Effects of exercise on breast cancer patients and survivors: a systematic review and meta-analysis. *Canadian Medical Association Journal.* 2006;175(1):34-41. Inappropriate Outcomes

179. McNeely MLC, K.S. Exercise programs for cancer-related fatigue: evidence and clinical guidelines. *Journal of the National Comprehensive Cancer Network.* 2010;8(8):945-953. Inappropriate Study Design

180. McNeely ML. Exercise rehabilitation for breast and head and neck cancer survivors. *ProQuest Dissertations and Theses.* 2007:242. Inappropriate Outcomes

181. McNeil C. No rest for fatigue researchers. *Journal of the National Cancer Institute.* 2008;100(16):1129-1131. Inappropriate Study Design

182. McTiernan A. Physical activity after cancer: physiologic outcomes. *Cancer Investigation.* 2004;22(1):68-81. Inappropriate Study Design

183. Meneses-Echavez JFG-J, E.; Correa, J.E.; Ramirez-Velez, R. Supervised physical activity interventions in the management of cancer-related fatigue: A systematic review. *Nutricion Hospitalaria.* 2014;30(3):486-497. Inappropriate Study Design

184. Meneses-Echavez JFG-J, E.; Correa-Bautista, J.E.; Valle, J.S.; Ramirez-Velez, R. [Effectiveness of physical exercise on fatigue in cancer patients during active treatment: a systematic review and meta-analysis]. *Cad Saude Publica.* 2015;31(4):667-681. Inappropriate Population

185. Meneses JFV, R.R. Center-based exercise on cancer-related fatigue in breast cancer survivors during active treatment: A meta-analysis. *Annals of Oncology.* 2014;25. Inappropriate Study Design

186. Meriggi F. Cancer-related fatigue: still an enigma to be solved quickly. *Reviews on Recent Clinical Trials.* 2014;9(4):267-270. Inappropriate Study Design

187. Mewes JCS, L.M.G.; Lizerman, M.J.; van Harten, W.H. Effectiveness of multidimensional cancer survivor rehabilitation and cost-effectiveness of cancer rehabilitation in general: A systematic review. *Oncologist.* 2012;17(12):1581-1593. Inappropriate Study Design

188. Minton OB, A.; Barsevick, A.; Cramp, F.; Goedendorp, M.; Mitchell, S.A.; Stone, P.C. Cancer-related fatigue and its impact on functioning. *Cancer.* 2013;119 Suppl 11:2124-2130. Inappropriate Study Design

189. Minton OJ, F.; Jane, M. The role of behavioural modification and exercise in the management of cancer-related fatigue to reduce its impact during and after cancer treatment. *Acta Oncologica.* 2015;54(5):581-586. Inappropriate Study Design

190. Mishra SIS, R.W.; Snyder, C.; Geigle, P.M.; Berlanstein, D.R.; Topaloglu, O. Exercise interventions on health-related quality of life for people with cancer during active treatment. *Cochrane Database of Systematic Reviews.* 2012(8):CD008465. Inappropriate Study Design

191. Mishra SIS, R.W.; Snyder, C.; Geigle, P.; Gotay, C. Are exercise programs effective for improving health-related quality of life among cancer survivors? A systematic review and meta-analysis. *Oncology Nursing Forum.* 2014;41(6):E326-E342. Inappropriate Study Design

192. Mishra SIS, R.W.; Snyder, C.; Geigle, P.; Gotay, C. The effectiveness of exercise interventions for improving health-related quality of life from diagnosis through active cancer treatment. *Oncology Nursing Forum.* 2015;42(1):E33-E53. Inappropriate Study Design

193. Mitchell SAB, A.M. Cancer-related fatigue: The evidence base for assessment and management. *Cancer Journal.* 2006;12(5):374-387. Inappropriate Study Design

194. Mitchell SAB, S.L.; Hood, L.E.; Moore, K.; Tanner, E.R. Putting evidence into practice: evidence-based interventions for fatigue during and following cancer and its treatment. *Clinical Journal of Oncology Nursing.* 2007;11(1):99-113. Inappropriate Study Design

195. Mitchell SA. Cancer-related fatigue: state of the science. *Physical Medicine and Rehabilitation.* 2010;2(5):364-383. Inappropriate Study Design

196. Mock VO, M. Current management of fatigue and anemia in patients with cancer. *Seminars in Oncology Nursing.* 2003;19(4 Suppl 2):36-41. Inappropriate Study Design

197. Mock V. Evidence-based treatment for cancer-related fatigue. *Journal of the National Cancer Institute Monographs.* 2004(32):112-118. Inappropriate Study Design

198. Mohamad HM, G.; Haseen, F.; Ndow, J.; Craig, L.C.A.; Heys, S.D. The effect of dietary and exercise interventions on body weight in prostate cancer patients: A systematic review. *Nutrition and Cancer.* 2015;67(1):43-60. Inappropriate Intervention

199. Montazeri A. Health-related quality of life in breast cancer patients: a bibliographic review of the literature from 1974 to 2007. *Journal of Experimental and Clinical Cancer Research.* 2008;27:32. Inappropriate Study Design

200. Morrow GRA, P.L.; Hickok, J.T.; Roscoe, J.A.; Matteson, S. Fatigue associated with cancer and its treatment. *Supportive Care in Cancer.* 2002;10(5):389-398. Inappropriate Study Design

201. Mustian KA, C.; Piper, B.; Smith, T.; Sprod, L.; Scarpato, J.; Leach, C.; Peppone, L.; Palesh, O.; Jing, L.; Mohr, D.; Spring, B.; Berendsen, M.; Heckler, C.; Miller, S. A meta-analytic comparison of exercise, psychological, exercise combined with psychological and pharmaceutical interventions for cancer-related fatigue. *Annals of Behavioral Medicine.* 2012;43:S54-S54. Inappropriate Study Design

202. Mustian KMM, G.R.; Carroll, J.K.; Figueroa-Moseley, C.D.; Jean-Pierre, P.; Williams, G.C. Integrative nonpharmacologic behavioral interventions for the management of cancer-related fatigue. *Oncologist.* 2007;12 Suppl 1:52-67. Inappropriate Study Design

203. Nail LM. Fatigue in patients with cancer. *Oncology Nursing Forum.* 2002;29(3):537. Inappropriate Study Design

204. Nguyen PLA, S.M.; Basaria, S.; D'Amico, A.V.; Kantoff, P.W.; Keating, N.L.; Penson, D.F.; Rosario, D.J.; Tombal, B.; Smith, M.R. Adverse effects of androgen deprivation therapy and strategies to mitigate them. *European Urology.* 2015;67(5):825-836. Inappropriate Intervention

205. Oerlemans SM, F.; Nijziel, M.R.; Lybeert, M.; van de Poll-Franse, LV. The impact of treatment, socio-demographic and clinical characteristics on health-related quality of life among Hodgkin's and non-Hodgkin's lymphoma survivors: a systematic review. *Annals of Hematology.* 2011;90(9):993-1004. Inappropriate Study Design

206. Oldervoll LMK, S.; Hjermstad, M.; Lund, J.A.; Loge, J.H. Physical exercise results in the improved subjective well-being of a few or is effective rehabilitation for all cancer patients? *European Journal of Cancer.* 2004;40(7):951-962. Inappropriate Study Design

207. Ortega FBR, J.R.; Castillo, M.J.; Sjostrom, M. Physical fitness in childhood and adolescence: a powerful marker of health. *International Journal of Obesity.* 2008;32(1):1-11. Inappropriate Population

208. Ostergren PBK, C.; Bennedbaek, F.N.; Faber, J.; Sonksen, J.; Fode, M. The use of exercise interventions to overcome adverse effects of androgen deprivation therapy. *Nature Reviews Urology.* 2016;13(6):353-364. Inappropriate Outcomes

209. Ozalevli S. Impact of physiotherapy on patients with advanced lung cancer. *Chron Respir Dis.* 2013;10(4):223-232. Inappropriate Study Design

210. Paramanandam VSD, V. Exercise for the management of cancer-related fatigue in lung cancer: a systematic review. *European Journal of Cancer Care.* 2015;24(1):4-14. Inappropriate Study Design

211. Parsons JAD, A.M. Rehabilitation and quality-of-life issues in patients with extremity soft tissue sarcoma. *Current Treatment Options in Neurology.* 2004;5(6):477-488. Inappropriate Population

212. Pavic MS, P.; Rousset, H.; Debourdeau, P. [Management of cancer-related fatigue]. *Presse Med.* 2008;37(6 Pt 1):957-966. Inappropriate Study Design

213. Payne CW, P.J.; Martin, S. Interventions for fatigue and weight loss in adults with advanced progressive illness. *Cochrane Database of Systematic Reviews.* 2012;1:CD008427. Inappropriate Study Design

214. Pedersen BKS, B. Evidence for prescribing exercise as therapy in chronic disease. *Scandinavian Journal of Medicine & Science in Sports.* 2006;16 Suppl 1:3-63. Inappropriate Study Design

215. Penedo FJS, N.; Dahn, J.R.; Gonzalez, J.S. Physical activity interventions in the elderly: cancer and comorbidity. *Cancer Investigation.* 2004;22(1):51-67. Inappropriate Study Design

216. Persoon SK, M.J.; van der Weiden, K.; Buffart, L.M.; Nollet, F.; Brug, J.; Chinapaw, M.J. Effects of exercise in patients treated with stem cell transplantation for a hematologic malignancy: a systematic review and meta-analysis. *Cancer Treatment Reviews.* 2013;39(6):682-690. Inappropriate Intervention

217. Pinto BMM, N.C. Exercise in the rehabilitation of breast cancer survivors. *Psycho-Oncology.* 1999;8(3):191-206. Inappropriate Study Design

218. Portenoy RKI, L.M. Cancer-related fatigue: guidelines for evaluation and management. *Oncologist.* 1999;4(1):1-10. Inappropriate Study Design

219. Potempa KM. Chronic fatigue. *Annu Rev Nurs Res.* 1993;11:57-76. Inappropriate Study Design

220. Prue GR, J.; Allen, J.; Gracey, J.; Cramp, F. Cancer-related fatigue: A critical appraisal. *European Journal of Cancer.* 2006;42(7):846-863. Inappropriate Study Design

221. Puetz TWH, M.P. Differential effects of exercise on cancer-related fatigue during and following treatment A meta-analysis. *American Journal of Preventive Medicine.* 2012;43(2):E1-E24. Inappropriate Population

222. Puetz TWH, M.P. Effects of exercise on cancer-related fatigue in patients during and following cancer treatment: A systematic review and meta-analysis of randomized controlled trials. *Annals of Behavioral Medicine.* 2012;43:S273-S273. Inappropriate Study Design

223. Radbruch LS, F.; Elsner, F.; Goncalves, J.F.; Loge, J.; Kaasa, S.; Nauck, F.; Stone, P. Fatigue in palliative care patients -- an EAPC approach. *Palliative Medicine.* 2008;22(1):13-32. Inappropriate Study Design

224. Rao AC, H.J. Symptom management in the elderly cancer patient: fatigue, pain, and depression. *Journal of the National Cancer Institute Monographs.* 2004(32):150-157. Inappropriate Study Design

225. Rao AVC, H.J. Fatigue in older cancer patients: etiology, assessment, and treatment. *Seminars in Oncology.* 2008;35(6):633-642. Inappropriate Study Design

226. Ream ER, A. From theory to practice: designing interventions to reduce fatigue in patients with cancer. *Oncology Nursing Forum.* 1999;26(8):1295-1303. Inappropriate Study Design

227. Reif Kd, Vries U.; Petermann, F. [What does really help against cancer-related fatigue? An overview of systematic reviews]. *Pflege.* 2012;25(6):439-457. Inappropriate Study Design

228. Rogers LQM, E.; Anton, P.M.; Courneya, K.S.; Vicari, S.; Hopkins-Price, P.; Verhulst, S.; Mocharnuk, R.; Hoelzer, K. Better exercise adherence after treatment for cancer (BEAT Cancer) study: rationale, design, and methods. *Contemporary Clinical Trials.* 2012;33(1):124-137. Inappropriate Study Design

229. Roine ER, R.P.; Rasanen, P.; Vuori, I.; Sintonen, H.; Saarto, T. Cost-effectiveness of interventions based on physical exercise in the treatment of various diseases: a systematic literature review. *International Journal of Technology Assessment in Health Care.* 2009;25(4):427-454. Inappropriate Population

230. Romanelli AB, A.; Magrone, G.; Pascoli, M.; Sterzi, S. Cancer-related fatigue: evaluation and treatment. *Rays.* 2004;29(4):453-455. Inappropriate Study Design

231. Romieu IT, M.; Ferrari, P.; Bignon, Y.J.; Antoun, S.; Berthouze-Aranda, S.; Bachmann, P.; Duclos, M.; Ninot, G.; Romieu, G.; Senesse, P.; Behrendt, J.; Balosso, J.; Pavic, M.; Kerbrat, P.; Serin, D.; Tredan, O.; Fervers, B. [Physical activity and cancer survival]. *Bulletin Du Cancer.* 2012;99(10):979-994. Inappropriate Study Design

232. Rosenthal TCM, B.A.; Pretorius, R.; Malik, K. Fatigue: an overview. *American Family Physician.* 2008;78(10):1173-1179. Inappropriate Study Design

233. Rutledge DND, J.A.; Cunningham, M. A process model for evidence-based literature syntheses. *Oncology Nursing Forum.* 2004;31(3):543-550. Inappropriate Study Design

234. Ryan JLC, J.K.; Ryan, E.P.; Mustian, K.M.; Fiscella, K.; Morrow, G.R. Mechanisms of cancer-related fatigue. *Oncologist.* 2007;12 Suppl 1:22-34. Inappropriate Study Design

235. Salakari MRJS, T.; Nurminen, R.; Pylkkanen, L. Effects of rehabilitation among patients with advances cancer: a systematic review. *Acta Oncologica.* 2015;54(5):618-628. Inappropriate Study Design

236. Sammut LW, M.; Patel, N. Physical activity and quality of life in head and neck cancer survivors: a literature review. *International Journal of Sports Medicine.* 2014;35(9):794-799. Inappropriate Study Design

237. Schmitz K. Physical activity and breast cancer survivorship. *Recent Results in Cancer Research.* 2011;186:189-215. Inappropriate Study Design

238. Schmitz KHH, J.; Courneya, K.S.; Masse, L.C.; Duval, S.; Kane, R. Controlled physical activity trials in cancer survivors: a systematic review and meta-analysis. *Cancer Epidemiology, Biomarkers & Prevention.* 2005;14(7):1588-1595. Inappropriate Study Design

239. Schmitz KHC, K.S.; Matthews, C.; Demark-Wahnefried, W.; Galvao, D.A.; Pinto, B.M.; Irwin, M.L.; Wolin, K.Y.; Segal, R.J.; Lucia, A.; Schneider, C.M.; von Gruenigen, V.E.; Schwartz, A.L. American College of Sports Medicine roundtable on exercise guidelines for cancer survivors. *Medicine and Science in Sports and Exercise.* 2010;42(7):1409-1426. Inappropriate Study Design

240. Schmitz KH. Exercise for secondary prevention of breast cancer: moving from evidence to changing clinical practice. *Cancer Prevention Research.* 2011;4(4):476-480. Inappropriate Study Design

241. Schneider CMD, C.A.; Roozeboom, M.; Carter, S.D. A model program: exercise intervention for cancer rehabilitation. *Integrative Cancer Therapies.* 2002;1(1):76-82. Inappropriate Study Design

242. Schwartz AL. Understanding and treating cancer-related fatigue. *Oncology.* 2007;21(11 Suppl Nurse Ed):30-34. Inappropriate Study Design

243. Schwartz AL. Fatigue in long-term cancer survivors. *Oncology.* 2009;23(8 Suppl):27, 33-27, 34. Inappropriate Study Design

244. Segal R. Physical functioning for prostate health. *Canadian Urological Association Journal.* 2014;8(7-8 Suppl 5):S162-S163. Inappropriate Study Design

245. Shabaruddin FHC, L.C.; Elliott, R.A.; Payne, K. A systematic review of utility values for chemotherapy-related adverse events. *Pharmacoeconomics.* 2013;31(4):277-288. Inappropriate Intervention

246. Shaiova L. The management of opioid-related sedation. *Current Pain and Headache Reports.* 2005;9(4):239-242. Inappropriate Study Design

247. Shannon VR. Role of pulmonary rehabilitation in the management of patients with lung cancer. *Current Opinion in Pulmonary Medicine.* 2010;16(4):334-339. Inappropriate Study Design

248. Silver JK. Rehabilitation in women with breast cancer. *Physical Medicine and Rehabilitation Clinics of North America.* 2007;18(3):521-537, x. Inappropriate Study Design

249. Sluman MAV, M.C.; van der Wall, E.E.; Westerveld, H.E. [Women and cardiovascular disease]. *Nederlands Tijdschrift Voor Geneeskunde.* 2006;150(37):2018-2022. Inappropriate Outcomes

250. Smith-Turchyn JR, J. A systematic review on the use of exercise interventions for individuals with myeloid leukemia. *Supportive Care in Cancer.* 2015;23(8):2435-2446. Inappropriate Intervention

251. Smith GFT, T.R. Primary care of the patient with cancer. *American Family Physician.* 2007;75(8):1207-1214. Inappropriate Study Design

252. Smith MR. Changes in body composition during hormonal therapy for prostate cancer. *Clinical Prostate Cancer.* 2003;2(1):18-21. Inappropriate Study Design

253. Smits AL, A.; Das, N.; Bekkers, R.; Massuger, L.; Galaal, K. The effect of lifestyle interventions on the quality of life of gynaecological cancer survivors A systematic review and meta-analysis. *Gynecologic Oncology.* 2015;139(3):546-552. Inappropriate Intervention

254. Soares WTE. Parameters, considerations and modulation of physical exercise programs for oncologic patients - A systematic review. *Revista Brasileira de Medicina do Esporte.* 2011;17(4):284-289. Inappropriate Study Design

255. Sola-Penna M. Metabolic regulation by lactate. *IUBMB Life.* 2008;60(9):605-608. Inappropriate Study Design

256. Sood AM, T.J. Cancer-related fatigue: an update. *Current Oncology Reports.* 2005;7(4):277-282. Inappropriate Study Design

257. Sorensen JCC, B.D.; Timpani, C.A.; Nurgali, K.; Hayes, A.; Rybalka, E. Mitochondria: Inadvertent targets in chemotherapy-induced skeletal muscle toxicity and wasting? *Cancer Chemotherapy and Pharmacology.* 2016. Inappropriate Study Design

258. Spathis AB, S.; Grove, S.; Hatcher, H.; Kuhn, I.; Barclay, S. Teenage and young adult cancer-related fatigue is prevalent, distressing, and neglected: It is time to intervene. A systematic literature review and narrative synthesis. *Journal of Adolescent and Young Adult Oncology.* 2015;4(1):3-17. Inappropriate Population

259. Speck RMC, K.S.; Masse, L.C.; Duval, S.; Schmitz, K.H. An update of controlled physical activity trials in cancer survivors: a systematic review and meta-analysis. *Journal of Cancer Survivorship-Research and Practice.* 2010;4(2):87-100. Inappropriate Study Design

260. Spence RRH, K.C.; Brown, W.J. Exercise and cancer rehabilitation: A systematic review. *Cancer Treatment Reviews.* 2010;36(2):185-194. Inappropriate Study Design

261. Spencer JCW, S.B. A systematic review of Motivational Interviewing interventions in cancer patients and survivors. *Patient Education and Counseling.* 2016;99(7):1099-1105. Inappropriate Intervention

262. St Pierre BAK, C.E.; Lindsey, A.M. Fatigue mechanisms in patients with cancer: effects of tumor necrosis factor and exercise on skeletal muscle. *Oncology Nursing Forum.* 1992;19(3):419-425. Inappropriate Study Design

263. Stasi RA, L.; Beccaglia, P.; Terzoli, E.; Amadori, S. Cancer-related fatigue: evolving concepts in evaluation and treatment. *Cancer.* 2003;98(9):1786-1801. Inappropriate Study Design

264. Steinberg AA, A.; Bailey, C.; Fu, J.B. The role of physical rehabilitation in stem cell transplantation patients. *Supportive Care in Cancer.* 2015;23(8):2447-2460. Inappropriate Study Design

265. Stevinson CL, D.A.; Fox, K.R. Exercise interventions for cancer patients: systematic review of controlled trials. *Cancer Causes & Control.* 2004;15(10):1035-1056. Inappropriate Study Design

266. Stone P. The measurement, causes and effective management of cancer-related fatigue. *International Journal of Palliative Nursing.* 2002;8(3):120-128. Inappropriate Study Design

267. Stone PCM, O. Cancer-related fatigue. *European Journal of Cancer.* 2008;44(8):1097-1104. Inappropriate Study Design

268. Storer TWM, R.; Travison, T.G. Muscle function, physical performance and body composition changes in men with prostate cancer undergoing androgen deprivation therapy. *Asian Journal of Andrology.* 2012;14(2):204-221. Inappropriate Study Design

269. Stout NLB, J.M.; Schmitz, K.H.; Andrews, K.; Hayes, S.C.; Campbell, K.L.; McNeely, M.L.; Soballe, P.W.; Berger, A.M.; Cheville, A.L.; Fabian, C.; Gerber, L.H.; Harris, S.R.; Johansson, K.; Pusic, A.L.; Prosnitz, R.G.; Smith, R.A. A prospective surveillance model for rehabilitation for women with breast cancer. *Cancer.* 2012;118(8 Suppl):2191-2200. Inappropriate Intervention

270. Strasser BS, K.; Wiskemann, J.; Ulrich, C.M. Impact of resistance training in cancer survivors: A meta-analysis. *Medicine and Science in Sports and Exercise.* 2013;45(11):2080-2090. Inappropriate Outcomes

271. Stricker CTD, D.; Hoyer, K.A.; Mock, V. Evidence-based practice for fatigue management in adults with cancer: exercise as an intervention. *Oncology Nursing Forum.* 2004;31(5):963-976. Inappropriate Study Design

272. Strong AK, S.G.; Reicherter, E.A. Recommended exercise protocol to decrease cancer-related fatigue and muscle wasting in patients with multiple myeloma - An evidence-based systematic review. *Topics in Geriatric Rehabilitation.* 2006;22(2):172-186. Inappropriate Study Design

273. Stubbe CEV, M. Complementary strategies for the management of radiation therapy side effects. *Journal of the Advanced Practitioner in Oncology.* 2013;4(4):219-231. Inappropriate Intervention

274. Su CXW, L.Q.; Grant, S.J.; Liu, J.P. Chinese herbal medicine for cancer-related fatigue: A systematic review of randomized clinical trials. *Complementary Therapies in Medicine.* 2014;22(3):567-579. Inappropriate Intervention

275. Swinburn PL, A.; Nathan, P.; Choueiri, T.K.; Cella, D.; Neary, M.P. Elicitation of health state utilities in metastatic renal cell carcinoma. *Current Medical Research and Opinion.* 2010;26(5):1091-1096. Inappropriate Intervention

276. Szczesniak KAO, P.; Ciecierska, A.; Sadkowski, T. Investigation of nutriactive phytochemical - gamma-oryzanol in experimental animal models. *Journal of Animal Physiology and Nutrition.* 2016;100(4):601-617. Inappropriate Intervention

277. Tatham BS, J.; Cheifetz, O.; Gillespie, J.; Snowden, K.; Temesy, J.; Vandenberk, L. The efficacy of exercise therapy in reducing shoulder pain related to breast cancer: a systematic review. *Physiotherapy Canada.* 2013;65(4):321-330. Inappropriate Study Design

278. Tavio MM, I.; Tirelli, U. [Tumor-correlated asthenia]. *Recenti Progressi in Medicini.* 2002;93(11):610-616. Inappropriate Study Design

279. Tavio MM, I.; Tirelli, U. Cancer-related fatigue (review). *International Journal of Oncology.* 2002;21(5):1093-1099. Inappropriate Study Design

280. Thomas RD, N. Lifestyle during and after cancer treatment. *Clinical Oncology : a Journal of the Royal College of Radiologists.* 2007;19(8):616-627. Inappropriate Study Design

281. Thorsen LC, K.S.; Stevinson, C.; Fossa, S.D. A systematic review of physical activity in prostate cancer survivors: outcomes, prevalence, and determinants. *Supportive Care in Cancer.* 2008;16(9):987-997. Inappropriate Study Design

282. Thune IS, S. [Is physical activity important in treatment and rehabilitation of cancer patients?]. *Tidsskrift for den Norske Lægeforening.* 2000;120(27):3302-3304. Inappropriate Study Design

283. To JG, A.S.; Jones, J.; Zhang, J.; Lowe, J.; Ezzat, S.; Gilbert, J.; Zahedi, A.; Segal, P.; Sawka, A.M. A systematic review of randomized controlled trials for management of persistent post-treatment fatigue in thyroid cancer survivors. *Thyroid.* 2015;25(2):198-210. Inappropriate Study Design

284. Tomlinson DD, C.; Beyene, J.; Sung, L. Effect of exercise on cancer-related fatigue: a meta-analysis. *American Journal of Physical Medicine & Rehabilitation.* 2014;93(8):675-686. Inappropriate Intervention

285. Tomlinson DZ, S.; Jones, H.; O'Sullivan, C.; Hesser, T.; Sung, L. The lived experience of fatigue in children and adolescents with cancer: a systematic review. *Supportive Care in Cancer.* 2016;24(8):3623-3631. Inappropriate Population

286. Tralongo PR, D.; Ferrau, F. Fatigue and aging. *Critical Reviews in Oncology/Hematology.* 2003;48(Suppl):S57-S64. Inappropriate Study Design

287. Tsaras GO-A, A.; Boateng, F.O.; Amoateng-Adjepong, Y. Complications associated with sickle cell trait: a brief narrative review. *American Journal of Medicine.* 2009;122(6):507-512. Inappropriate Outcomes

288. van Brussel MT, T.; Lucia, A.; van der Net, J.; Helders, P.J.M. Is physical fitness decreased in survivors of childhood leukemia? A systematic review. Leukemia; 2005, 2005. Inappropriate Population

289. van Weert EH-W, J.E.H.M.; May, A.M.; Korstjens, I.; Ros, W.J.G.; van der Schans, C.P. The development of an evidence-based physical self-management rehabilitation programme for cancer survivors. *Patient Education and Counseling.* 2008;71(2):169-190. Inappropriate Study Design

290. Vermaete NW, P.; Verhoef, G.; Gosselink, R. Physical activity, physical fitness and the effect of exercise training interventions in lymphoma patients: a systematic review. *Annals of Hematology.* 2013;92(8):1007-1021. Inappropriate Study Design

291. Visovsky CS, S.M. Cancer-related fatigue. *Online Journal of Issues in Nursing.* 2003;8(3):8. Inappropriate Study Design

292. Visovsky CD, C. Exercise and cancer recovery. *Online Journal of Issues in Nursing.* 2005;10(2):7. Inappropriate Study Design

293. Wanchai AA, J.M.; Stewart, B.R. Nonpharmacologic supportive strategies to promote quality of life in patients experiencing cancer-related fatigue: A systematic review. *Clinical Journal of Oncology Nursing.* 2011;15(2):203-214. Inappropriate Study Design

294. Watson TM, V. Exercise as an intervention for cancer-related fatigue. *Physical Therapy.* 2004;84(8):736-743. Inappropriate Study Design

295. Weis J. Cancer-related fatigue: prevalence, assessment and treatment strategies. *Expert Review of Pharmacoeconomics & Outcomes Research.* 2011;11(4):441-446. Inappropriate Study Design

296. Wick JYL, J. Fatigue: implications for the elderly. *The Consultant Pharmacist.* 2007;22(7):566-564, 576. Inappropriate Study Design

297. Wilmoth MCC, E.A.; Smith, S.C.; Davis, C. Fatigue, weight gain, and altered sexuality in patients with breast cancer: exploration of a symptom cluster. *Oncology Nursing Forum.* 2004;31(6):1069-1075. Inappropriate Outcomes

298. Winningham ML. Walking program for people with cancer. Getting started. *Cancer Nursing.* 1991;14(5):270-276. Inappropriate Study Design

299. Winningham MLN, L.M.; Burke, M.B.; Brophy, L.; Cimprich, B.; Jones, L.S.; Pickard-Holley, S.; Rhodes, V.; St, Pierre B.; Beck, S.; . Fatigue and the cancer experience: the state of the knowledge. *Oncology Nursing Forum.* 1994;21(1):23-36. Inappropriate Study Design

300. Winningham ML. Strategies for managing cancer-related fatigue syndrome: a rehabilitation approach. *Cancer.* 2001;92(4 Suppl):988-997. Inappropriate Intervention

301. Winters-Stone KMB, T.M. Review of exercise studies in prostate cancer survivors receiving androgen deprivation therapy calls for an aggressive research agenda to generate high-quality evidence and guidance for exercise as standard of care. *Journal of Clinical Oncology.* 2014;32(23):2518-2519. Inappropriate Study Design

302. Wiskemann JH, G. Physical exercise as adjuvant therapy for patients undergoing hematopoietic stem cell transplantation. *Bone Marrow Transplantation.* 2008;41(4):321-329. Inappropriate Study Design

303. Wiskemann JF-B, B. Capability of physical activity in prevention and therapy of chronic diseases. *Aktuelle Ernahrungsmedizin.* 2015;40(6):389-406. Inappropriate Study Design

304. Witt JM-E, D. Living with fatigue: managing cancer-related fatigue at home and in the workplace. *American Journal of Nursing.* 2002;102 Suppl 4:28-31. Inappropriate Study Design

305. Wolin KYR, J.R.; Tuchman, H.; Lucia, A. Exercise in adult and pediatric hematological cancer survivors: an intervention review. *Leukemia.* 2010;24(6):1113-1120. Inappropriate Population

306. Wolin KYS, A.L.; Matthews, C.E.; Courneya, K.S.; Schmitz, K.H. Implementing the exercise guidelines for cancer survivors. *Journal of Supportive Oncology.* 2012;10(5):171-177. Inappropriate Study Design

307. Wonders KYR, B.S. Trastuzumab and doxorubicin-related cardiotoxicity and the cardioprotective role of exercise. *Integrative Cancer Therapies.* 2009;8(1):17-21. Inappropriate Intervention

308. Wyrick KD, A. Exercise for the management of cancer-related fatigue. *American Family Physician.* 2009;80(7):689-690. Inappropriate Study Design

309. Yeh MLC, Y.C.; Hsu, M.Y.; Hsu, C.C. Quantifying psychological distress among cancer patients in interventions and scales: a systematic review. *Current Pain and Headache Reports.* 2014;18(3):399. Inappropriate Intervention

310. Zahner JM, J.; Karthaus, M. [Exhaustion and fatigue--a neglected problem in hematologic oncology]. *Wiener medizinische Wochenschrift.* 2001;151(3-4):89-93. Inappropriate Study Design

311. Zeng YCH, M.L.; Cheng, A.; Zhou, Y.; So, W.K.W. Meta-analysis of the effects of exercise intervention on quality of life in breast cancer survivors. *Breast Cancer.* 2014;21(3):262-274. Inappropriate Intervention

312. Zeng YCL, T.Z.; Xie, H.A.; Huang, M.L.; Cheng, A.S.K. Health benefits of qigong or tai chi for cancer patients: a systematic review and meta-analyses. *Complementary Therapies in Medicine.* 2014;22(1):173-186. Inappropriate Intervention

313. Zhang JY, K.H.; Tian, J.H.; Wang, C.M. Effects of yoga on psychologic function and quality of life in women with breast cancer: A meta-analysis of randomized controlled trials. *Journal of Alternative and Complementary Medicine.* 2012;18(11):994-1002. Inappropriate Intervention

314. Zheng YC, H.; Li, X.; Sun, Y. Pay attention to cardiac remodeling in cancer cachexia. *Supportive Care in Cancer.* 2016;24(7):3253-3259. Inappropriate Outcomes

315. Zhu GQZ, X.; Wang, Y.L.; Xiong, H.Z.; Zhao, Y.H.; Sun, F.Y. Effects of exercise intervention in breast cancer survivors: a meta-analysis of 33 randomized controlled trails. *Oncotargets and Therapy.* 2016;9:2153-2168. Inappropriate Intervention

316. Zwarts MJB, G.; van Engelen, B.G. Clinical neurophysiology of fatigue. *Clinical Neurophysiology.* 2008;119(1):2-10. Inappropriate Study Design
