# Supplementary material for: Exercise and cancer-related fatigue in adults: a systematic review of previous systematic reviews with meta-analyses
Source: BMC Cancer. 2017 Oct 23;17:693. doi: 10.1186/s12885-017-3687-5 (PMC5651567; doi:10.1186/s12885-017-3687-5)
Supplement: Supplementary file 3 — Item by item results using the AMSTAR assessment instrument. This file includes the results of the AMSTAR assessment for each item from each study. (DOCX 50 kb) [file 12885_2017_3687_MOESM3_ESM.docx]

**Additional file 3.** Item by item results using the AMSTAR assessment instrument.

| **Reference** | **A priori design provided?** | **Duplicate study selection and data extraction?** | **Comprehensive literature search?** | **Status of publication avoided?** | **List of included/ excluded studies provided?** | **Characteristics of studies provided?** | **Scientific quality assessed?** | **Scientific quality of studies used appropriately in conclusions?** | **Methods for combining studies appropriate?** | **Publication bias assessed?** | **Conflict of interest stated** | **Total score (%)^a^** |
| --- | --- | --- | --- | --- | --- | --- | --- | --- | --- | --- | --- | --- |
| Brown et al.[10] | yes | ca | yes | ca | no | yes | yes | yes | yes | yes | no | 78 |
| Carayol et al.[11] | yes | ca | ca | ca | no | yes | yes | yes | yes | yes | no | 75 |
| Carayol et al.[12] | yes | ca | yes | ca | no | yes | yes | yes | yes | yes | no | 78 |
| Cramer et al.[13] | yes | yes | yes | ca | no | yes | yes | yes | yes | na | no | 78 |
| Duijts et al.[16] | yes | ca | no | ca | no | yes | no | no | yes | yes | no | 44 |
| Fong et al.[17] | yes | yes | yes | ca | no | yes | yes | yes | yes | yes | no | 80 |
| Jacobsen et al.[18] | yes | ca | no | no | no | yes | yes | yes | ca | no | no | 44 |
| Kangas et al.[19] | yes | yes | no | no | no | yes | yes | yes | yes | yes | no | 64 |
| Meneses-Echavez et al.[24] | yes | yes | ca | yes | no | yes | yes | yes | yes | yes | no | 80 |
| Meneses-Echavez et al.[25] | yes | yes | ca | yes | no | yes | yes | yes | yes | yes | no | 80 |
| Meneses-Echavez et al.[26] | yes | ca | yes | yes | no | yes | yes | yes | yes | no | no | 70 |
| Tian et al.[31] | yes | ca | no | ca | no | yes | yes | yes | yes | no | no | 56 |
| Van Haren et al.[33] | yes | no | yes | no | no | yes | yes | yes | yes | yes | no | 64 |
| Van Vulpen et al.[34] | yes | yes | ca | ca | no | yes | yes | yes | yes | yes | no | 78 |
| Velthuis et al.[35] | yes | yes | ca | ca | no | yes | yes | yes | yes | no | no | 67 |
| Zou et al.[36] | yes | ca | ca | no | no | yes | yes | yes | yes | yes | no | 67 |

Notes: ca, can’t answer; na, not applicable; Possible responses were “Yes”, “No”, “Can’t Answer”, “Not Applicable”. “Can’t Answer” chosen when item is relevant but not described; “Not Applicable” chosen when item is not relevant (for example, insufficient number of studies to assess publication bias);^a^,scores adjusted for “can’t answer’ and “not applicable” responses.
