# Supplementary material for: Exercise and cancer-related fatigue in adults: a systematic review of previous systematic reviews with meta-analyses
Source: BMC Cancer. 2017 Oct 23;17:693. doi: 10.1186/s12885-017-3687-5 (PMC5651567; doi:10.1186/s12885-017-3687-5)
Supplement: Supplementary file 4 — Post-treatment standardized mean difference (SMD) effect sizes for CRF from included meta-analyses. This file includes the overall post-treatment changes in CRF from included meta-analyses. (DOCX 57 kb) [file 12885_2017_3687_MOESM4_ESM.docx]

**Additional file 4.** Post-treatment standardized mean difference (SMD) effect sizes for CRF from included meta-analyses.

| Reference/Group | ES/Participants (No.) | SMD (95% CI) | Z(p) | Q(p) | *I^2^* ^(%)^ | $\tau$^2^ | PI (95%) |
| --- | --- | --- | --- | --- | --- | --- | --- |
| Brown et al.[10] |  |  |  |  |  |  |  |
| - All studies | 48/3254 | -0.31 (-0.40, -0.22) | **-6.6(<0.001)*** | **93.4(<0.001)**** | 50 | 0.04 | -0.73, 0.11 |
| - Breast | 28/1474 | -0.39 (-0.51, -0.27) | **-6.3(<0.001)*** | **47.2(<0.001)**** | 42 | 0.04 | -0.82, 0.03 |
| - Prostate | 5/400 | -0.42 (-0.57, -0.27) | **-5.5(<0.001)*** | 3.2(0.53) | 0 | 0.00 | **-0.66,-0.18\|** |
| - Lymphoma | 4/227 | -0.19 (-0.43, 0.03) | -1.6(0.10) | 2.3(0.51) | 0 | 0.00 | na |
| Carayol et al.[11] |  |  |  |  |  |  |  |
| - Breast cancer | 13/-- | -0.28 (-0.54, -0.03) | **-2.2(0.03)*** | **43.0(<0.001)**** | 72 | 0.15 | -1.18,0.62 |
| Carayol et al.[12] |  |  |  |  |  |  |  |
| - Breast cancer^a^ | 20/1696 | -0.11 (-0.20, -0.01) | **-2.3(0.03)*** | 11.5(0.91) | 0 | 0.00 | **-0.21,-0.01\|** |
| Cramer et al.[13] |  |  |  |  |  |  |  |
| - Colorectal cancer | 3/157 | -0.18 (-0.59, 0.22) | -0.9(0.38) | 2.7(0.26) | 27 | 0.04 | na |
| Duijts et al.[16] |  |  |  |  |  |  |  |
| - Exercise only | 10/1002 | -0.35 (-0.53, -0.10) | **-2.9(<0.004)*** | **30.7(<0.00)**** | 71 | 0.08 | -1.02,0.39 |
| Fong et al.[17] ^b^ |  |  |  |  |  |  |  |
| - All studies^c^ | 13/1042 | -0.20 (-0.37, -0.04) | **-2.4(0.02)*** | **19.3(0.08)**** | 38 | 0.03 | -0.64,0.24 |
| - FACT | 8/758 | -0.03 (-0.18, 0.11) | -0.4(0.68) | 3.7(0.81) | 0 | 0 | na |
| - PFS | 3/101 | -0.44 (-0.84, -0.05) | **-2.0(0.05)*** | 0.9(0.65) | 0 | 0 | -3.01,2.13 |
| - EORTC^d^ | 2/183 | -0.61 (-0.91, -0.32) | **-4.1(<0.001)*** | 0.7(0.3) | 0 | 0 | -- |
| Jacobsen et al.[18] |  |  |  |  |  |  |  |
| - All studies | 10/737 | -0.08 (-0.23, 0.07) | -1.1(0.29) | 9.2(0.42) | 2 | -- | na |
| - Breast cancer | 4/209 | -0.12 (-0.30, 0.15) | -1.1(0.29) | **9.6(0.02)**** | 69 | -- | na |
| - Other cancers | 6/528 | -0.06 (-0.24, 0.11) | -0.7(0.50) | 8.5(0.13) | 41 | -- | na |
| - Home-based exercise | 6/494 | -0.04 (-0.22, 0.14) | -0.4(0.66) | 7.6(0.18) | 34 | -- | na |
| - Supervised exercise | 4/243 | -0.16 (-0.41, 0.09) | -1.3(0.21) | 1.1(0.78) | 0 | -- | na |
| Kangas et al.[19] |  |  |  |  |  |  |  |
| - All studies | 16/1001 | -0.42 (-0.60, -0.23)* | **-4.5(<0.001)*** | -- | -- | -- | -- |
| Meneses-Echavez et al.[24] |  |  |  |  |  |  |  |
| - All studies | 9/772 | -0.29 (-0.50, -0.09) | **-2.8(0.005)*** | **15.0(0.06)**** | 47 | 0.04 | -0.84,0.26 |
| - Active treatment | 6/-- | -0.33 (-0.63, -0.04) | **-2.2(0.03)*** | **13.9(0.02)**** | 65 | 0.08 | -1.22,0.56 |
| - After treatment | 3/-- | -0.25 (-0.56, 0.05) | -1.6(0.10) | 1.1(0.59) | 0 | 0 | na |
| - Aerobic & strength | 7/-- | -0.35 (-0.62, -0.08) | **-2.6(0.01)*** | **14.7(0.02)**** | 59 | 0.07 | -1.12,0.42 |
| - Strength only | 2/-- | -0.17 (-0.50, 0.15) | -1.0(0.30) | 0.1(0.78) | 0 | 0 | na |
| Meneses-Echavez et al.[25] |  |  |  |  |  |  |  |
| - All studies | 9/1016 | -0.51 (-0.81, -0.22) | **-3.4(0.001)*** | **31.7(0.0001)**** | 75 | 0.14 | -1.45,0.43 |
| -Strength training included | 6/-- | -0.41 (-0.76, -0.06) | **-2.3(0.02)*** | **13.8(0.02)**** | 64 | 0.12 | -1.48,0.66 |
| - Active treatment | 6/-- | -0.66 (-1.09, -0.24) | **-3.1(0.002)*** | **30.6(<0.001)**** | 79 | 0.21 | -2.07,0.74 |
| - After treatment | 3/-- | -0.26 (-0.56, 0.05) | -1.7(0.10) | 1.1(0.59) | 0 | 0 | na |
| Meneses-Echavez et al.[26] |  |  |  |  |  |  |  |
| - All studies^e^ | 11/1427 | -0.31 (-0.55, -0.08) | **-2.6(0.009)*** | **40.4(<0.001)**** | 75 | 0.11 | -1.11,0.49 |
| - Aerobic exercise^e^ | 5/803 | -0.02 (-0.27, 0.22) | -0.1(0.87) | **8.0(0.09)**** | 50 | 0.04 | na |
| - Aerobic & strength | 2/193 | -0.41 (-0.70, -0.13) | **-2.8(0.005)*** | 0.5(0.47) | 0 | 0 | -- |
| - Aerobic, strength, stretch | 4/431 | -0.67 (-1.17, -0.17) | **-2.6(0.009)*** | **15.8(0.001)**** | 81 | 0.20 | -2.88,1.55 |
| Tian et al.[31] |  |  |  |  |  |  |  |
| - All studies | 36/2830 | -0.22 (-0.38, -0.04) | **-2.5(0.05)*** | **158.6(<0.001)**** | 78 | 0.19 | -1.12,0.68 |
| van Haren et al.[33] |  |  |  |  |  |  |  |
| - All studies | 2/115 | -0.53 (-0.91, -0.16) | **-2.8(0.005)*** | 0.1(0.73) | 0 | 0 | -- |
| van Vulpen et al.[34] |  |  |  |  |  |  |  |
| - General fatigue | 4/-- | -0.22 (-0.38, -0.05) | **-2.0(0.04)*** | 0.4(0.94) | 0 | 0 | -0.58,0.14 |
| - Physical fatigue | 6/-- | -0.35 (-0.49, -0.21) | **-5.0(<0.001)*** | 4.1(0.53) | 0 | 0 | **-0.55, -0.15\|** |
| - Affective fatigue | 2/-- | -0.06 (-0.31, 0.20) | -0.5(0.64) | 0.2(0.67) | 0 | 0 | na |
| - Cognitive fatigue | 6/-- | -0.06 (-0.20, 0.08) | -0.6(0.53) | 1.5(0.91) | 0 | 0 | na |
| - Reduced activity | 4/-- | -0.22 (-0.38, -0.05) | **-2.6(0.008)*** | 1.7(0.64) | 0 | 0 | -0.58,0.14 |
| - Reduced motivation | 4/-- | -0.18 (-0.35, -0.01) | **-2.1(0.04)*** | 1.7(0.64) | 0 | 0 | -0.55, 0.19 |
| Velthuis et al.[35] |  |  |  |  |  |  |  |
| - Breast (all studies) | 8/674 | -0.29 (-0.52, -0.06) | **-2.5(0.01)*** | **12.6(0.08)**** | 44 | 0.04 | -0.87, 0.29 |
| - Breast (home-based) | 2/128 | -0.10 (-0.45, 0.25) | -0.6(0.57) | 0.1(0.77) | 0 | 0 | na |
| - Breast (supervised, aerobic) | 3/340 | -0.30(-0.51, -0.09) | **-2.7(0.006)*** | 1.5(0.46) | 0 | 0 | -1.66,1.06 |
| - Breast (supervised aerobic & strength | 2/57 | -1.04 (-2.80, 0.71) | -1.2(0.25) | **7.4(0.006)**** | 87 | 0 | na |
| - Prostate (all studies) | 5/371 | -0.32 (-0.59, -0.05) | **-2.4(0.02)*** | 6.2(0.18) | 36 | 0.03 | -1.05, 0.41 |
| - Prostate (supervised aerobic) | ^2/98^ | -0.76 (-1.93, 0.42) | -1.3(0.21) | **4.8(0.03)**** | 79 | 0.58 | na |
| - Prostate (supervised strength) | 2/208 | -0.20 (-0.47, 0.07) | -1.4(0.15) | 0.5(0.49) | 0 | 0 | na |
| Zou et al.[36] |  |  |  |  |  |  |  |
| - RPFS (all studies) ^f^ | 6/371 | -0.72 (-1.40, -0.04) | **-2.1(0.4)*** | **43.4(<0.001)**** | 89 | 0.61 | -3.11,1.67 |
| - FACIT-F (all studies) ^f^ | 6/643 | -0.27 (-0.70, 0.16) | -1.2(0.2) | **31.2(<0.001)**** | 84 | 0.23 | na |
| - RPFS (Asian) ^f^ | 3/262 | -1.05 (-2.13, 0.02) | -1.9(0.05) | **32.0 (<0.001)**** | 94 | 0.85 | na |
| - RPFS (Caucasian) ^f^ | 3/109 | -0.29 (-0.68, 0.09) | -1.5(0.14) | 0.6(0.74) | 0 | 0 | na |
| - FACIT-F (Caucasian) | 5/571 | -0.04 (-0.35, 0.26) | -0.3(0.78) | **10.5(0.03)**** | 62 | 0.07 | na |
| - RPFS (<8 weeks) ^f^ | 4/334 | -0.85 (-1.71, 0.02) | -1.95(0.05) | **92.8(<0.001)**** | 93 | 0.72 | na |
| - RPFS (>8 weeks) | 2/37 | -0.41 (-1.09, 0.27) | -1.2(0.24) | 0.45(0.50) | 0 | 0 | na |
| - FACIT-F (< 8 weeks) | 2/110 | -0.76 (-1.67, 0.15) | -1.65(0.10) | **4.9(0.03)**** | 80 | 0.34 | na |
| - FACIT-F (> 8 weeks) | 4/533 | -0.01 (-0.35, 0.33) | -0.07(0.95) | **9.5(0.02)**** | 69 | 0.08 | na |

Notes: No., Number; ES, effect size; SMD, standardized mean difference effect size; 95% CI, 95% confidence intervals; Z(p), Z-value and probability value for Z; Q(p), Cochran’s Q statistic and associated alpha (p) value for Q; *I^2^,* I-squared statistic for inconsistency; $\tau$^2^, tau-squared; PI, prediction intervals, based on a random-effects model; --, insufficient data available; na, not applicable because SMD results were not statistically significant; *, statistically significant at two-tailed alpha value <0.05; **, statistically significant at two-tailed alpha value <0.10; |, non-overlapping 95% confidence intervals; **boldfaced** items are statistically significant; ^a^, aerobic and/or resistance training only studies; ^b^, data initially reported using the original metric from each instrument but converted to SMD effect size for comparison purposes; ^c^, results not pooled in original meta-analysis; ^d^, not reported as statistically significant using the original metric; ^e^, results corrected for data entry error in original meta-analysis; ^f^, based on random versus fixed-effect model reported by authors; EORTC, European Organization for Research and Treatment of Cancer; FACIT-F, Functional Assessment of Chronic Illness Treatment-Fatigue; FACT, Functional Assessment of Cancer Therapy; PFS, Piper Fatigue Scale; RPFS, Revised Piper Fatigue Scale.
