# Supplementary material for: Exercise and cancer-related fatigue in adults: a systematic review of previous systematic reviews with meta-analyses
Source: BMC Cancer. 2017 Oct 23;17:693. doi: 10.1186/s12885-017-3687-5 (PMC5651567; doi:10.1186/s12885-017-3687-5)
Supplement: Supplementary file 5 — NNT and percentile improvements in CRF. This file includes data on NNT and percentile improvements for statistically significant changes in CRF. (DOCX 48 kb) [file 12885_2017_3687_MOESM5_ESM.docx]

**Additional file 5**. NNT and percentile improvements in CRF.

| Reference | NNT (95% CI) | U_3_ Index (95% CI)^a^ (Percentile Improvement) |
| --- | --- | --- |
| Brown et al.[10] |  |  |
| - All cancers | 6 (5,8) | 12.2 (8.6, 15.7) |
| - Breast cancer | 5 (4,7) | 15.2 (10.6, 19.6) |
| - Prostate cancer | 4 (3,7) | 16.3 (10.6, 21.6) |
| Carayol et al.[11] |  |  |
| - Breast cancer | 6 (3, 63) | 11.2 (1.1, 20.6) |
| Carayol et al.[12] |  |  |
| - Breast cancer^a^ | 16 (8, 167) | 4.4 (0.4, 7.9) |
| Duijts et al.[16] |  |  |
| - Exercise only | 6 (3, 18) | 12.4 (3.9, 20.3) |
| Fong et al.[17] |  |  |
| - All studies | 9 (5, 45) | 8.1 (1.6, 14.4) |
| - PFS | 4 (2, 36) | 17.0 (1.8, 30) |
| - EORTC | 3 (2, 6) | 22.9 (12.6, 31.9) |
| Kangas et al.[19] |  |  |
| - All studies | 4 (5, 8) | 16.1 (9.1, 22.5) |
| Meneses-Echavez et al.[24] |  |  |
| - All studies | 6 (4, 22) | 11.4 (3.2, 19.1) |
| - Active treatment | 5 (3, 45) | 12.9 (1.6, 23.6) |
| - Aerobic & strength | 5 (3, 22) | 13.7 (3.2, 23.2) |
| Meneses-Echavez et al.[25] |  |  |
| - All studies | 4 (2, 8) | 19.5 (8.5, 29) |
| -Strength training included | 4 (2, 29) | 15.9 (2.3, 27.7) |
| - Active treatment | 3 (2, 7) | 24.6 (9.4, 36.1) |
| Meneses-Echavez et al.[26] |  |  |
| - All studies | 6 (3, 22) | 12.2 (3.2, 20.9) |
| - Aerobic & strength | 4 (3, 14) | 15.9 (5.2, 25.8) |
| - Aerobic, strength, stretch | 3 (2, 10) | 24.9 (6.7, 37.9) |
| Tian et al.[31] |  |  |
| - All studies | 8 (5, 45) | 8.7 (1.6, 15.2) |
| Van Haren et al.[33] |  |  |
| - All studies | 3 (2,11) | 20.2 (6.4, 31.9) |
| Van Vulpen et al.[34] |  |  |
| - General fatigue | 8 (5, 36) | 8.7 (2.0, 14.8) |
| - Physical fatigue | 5 (4, 8) | 13.7 (8.3, 18.8) |
| - Reduced activity | 8 (5, 36) | 8.7 (2.0, 14.8) |
| - Reduced motivation | 10 (5, 167) | 7.1 (0.4, 13.7) |
| Velthuis et al.[35] |  |  |
| - Breast (all studies) | 6 (4, 29) | 11.4 (2.4, 19.8) |
| - Breast (supervised aerobic) | 6 (4, 20) | 11.8 (3.6, 19.5) |
| - Prostate (all studies) | 6 (3, 36) | 12.6 (2.0, 22.2) |
| Zou et al.[36] |  |  |
| - RPFS Scores | 3 (1, 45) | 26.4 (1.6, 41.9) |

Notes: NNT, number needed to-treat, calculated from SMD and 95% confidence intervals for SMD; 95% CI, 95% confidence intervals; ^a^, aerobic and/or resistance training only; EORTC, European Organization for Research and Treatment of Cancer; PFS, Piper Fatigue Scale; RPFS, Revised Piper Fatigue Scale.
